# Tick Densities and Infection Prevalence on Coastal Islands in Massachusetts, USA: Establishing a Baseline

**DOI:** 10.3390/insects14070628

**Published:** 2023-07-12

**Authors:** Allison A. Snow, Patrick Pearson, Guang Xu, David N. Allen, Roberto Santamaria, Stephen M. Rich

**Affiliations:** 1 Department of Evolution, Ecology, and Organismal Biology, Ohio State University, Columbus, OH 43210, USA; 2 Department of Biology, University of Massachusetts, Amherst, MA 01003, USA; 3 Laboratory of Medical Zoology, Department of Microbiology, University of Massachusetts, Amherst, MA 01003, USA; pbpearson@umass.edu (P.P.); gxu@umass.edu (G.X.); smrich@umass.edu (S.M.R.); 4 Department of Biology, Middlebury College, Middlebury, VT 05753, USA; dallen@middlebury.edu; 5 Nantucket Board of Health, Nantucket, MA 02554, USA; rsantamaria@nantucket-ma.gov

**Keywords:** tick-borne pathogen, blacklegged tick, lone star tick, Lyme disease, anaplasmosis, babesiosis

## Abstract

**Simple Summary:**

People who use hiking trails may be exposed to blacklegged ticks (*Ixodes scapularis*, also known as deer ticks), some of which are infected with the pathogens that cause Lyme disease, anaplasmosis, and babesiosis. In areas that also have lone star ticks (*Amblyomma americanum*), an added concern is acquiring the alpha-gal red meat allergy. Here, we describe an example of how such tick-related risks can be assessed at the scale of a local community, while providing a baseline for further monitoring. We used drag sampling along public trails to quantify tick abundance in June 2020–2022 at 12 study sites in the town of Nantucket, Massachusetts, USA. One of these sites was located on nearby Tuckernuck Island. Blacklegged nymphs were common at sites with moist deciduous woodlands and rare in open grasslands. For several sites, we carried out pathogen testing and found that ~10–20% of blacklegged nymphs on Nantucket were infected with the bacterium that causes Lyme disease. Lone star ticks were extremely common on Tuckernuck Island and rare on Nantucket Island, where they are expected to become more widespread in the future. Both tick species represent a significant threat to public health and mitigating their impact is an ongoing challenge.

**Abstract:**

Tick-borne diseases and a tick-induced red meat allergy have become increasingly common in the northeastern USA and elsewhere. At the scale of local communities, few studies have documented tick densities or infection levels to characterize current conditions and provide a baseline for further monitoring. Using the town of Nantucket, MA, as a case study, we recorded tick densities by drag sampling along hiking trails in nature preserves on two islands. Nymphal blacklegged ticks (*Ixodes scapularis* Say) were most abundant at shadier sites and least common in grasslands and scrub oak thickets (*Quercus ilicifolia*). Lone star ticks (*Amblyomma americanum* L.) were common on Tuckernuck Island and rare on Nantucket Island, while both tick species were more numerous in 2021 compared to 2020 and 2022. We tested for pathogens in blacklegged nymphs at five sites over two years. In 2020 and 2021, infection levels among the four Nantucket Island sites averaged 10% vs. 19% for *Borrelia burgdorferi*, 11% vs. 15% for *Babesia microti,* and 17% (both years) for *Anaplasma phagocytophilum*, while corresponding levels were significantly greater on Tuckernuck in 2021. Our site-specific, quantitative approach represents a practical example of how potential exposure to tick-borne diseases can be monitored on a local scale.

## 1. Introduction

Lyme disease is the most common tick-borne disease in the USA, while other tick-borne diseases such as babesiosis and anaplasmosis are increasing in frequency [1,2,3,4]. Infected blacklegged ticks (*Ixodes scapularis*) carry the bacterial pathogen that causes Lyme disease, *Borrelia burgdorferi,* and other disease agents, including *Babesia microti* (babesiosis), *Anaplasma phagocytophilum* (anaplasmosis; formerly human granulocytic ehrlichiosis), *Borrelia miyamotoi* (relapsing fever), and Powassan virus [5,6]. Another public health concern in the eastern USA is the spread of lone star ticks (*Amblyomma americanum*), which are expanding their range northward and often co-occur with blacklegged ticks [7,8,9,10]. Although primarily a serious nuisance species, lone star ticks can transmit several disease agents and can cause the alpha-gal “red meat” allergy in people [11,12,13]. 

Efforts to understand the underlying causes of tick-borne disease transmission involve studies of tick abundance and infection status, as well as the roles that local wildlife species play as bloodmeal hosts for ticks and as reservoirs in the pathogens’ life cycles [14,15,16]. Blacklegged ticks typically have a 2-year life cycle and require a single bloodmeal at each active stage—as larvae in late summer, nymphs the following spring or summer, and adults in fall or the following spring [6]. Larvae and nymphs feed on a range of vertebrate hosts, including white-footed mice (*Peromyscus leucopus*), eastern chipmunks (*Tamias striatus*), other rodents, shrews (*Blarina brevicauda*, *Sorex cinereus*), ground-foraging birds, and deer (*Odocoileus virginianus*), while adult females feed primarily on deer [17,18,19]. Most cases of Lyme disease, babesiosis, and anaplasmosis are due to bites from nymphs, which are so small that they may not be noticed when taking a bloodmeal [20,21]. In contrast to blacklegged ticks, lone star nymphs as well as adults seek bloodmeals during early summer, larvae emerge in summer/early fall, and all three life stages feed primarily on deer [22,23]. 

Many large-scale surveys of human-biting ticks have been carried out across states or regions [24,25,26,27,28,29], but relatively few published studies focus on local towns or counties [15,30,31,32]. Although aggregated data on tick abundances and infection levels across states or regions are useful for documenting large-scale patterns of entomological risk, these data can be problematic for characterizing community-level conditions, especially when sampling efforts are spread over a large, heterogeneous area [33]. 

In this study, we sought to quantify tick abundance and infection status at the scale of a local residential community using two islands that comprise the town of Nantucket, MA, as a case study. Our findings document current conditions and provide a baseline for tracking changes over time, for example in response to extreme weather conditions, changes in host species abundances, or the arrival of new tick species and tick-borne pathogens. We focused on blacklegged ticks and lone star ticks, which are common on Tuckernuck Island but rare on Nantucket Island. We designed the study to rely on a small field crew to record tick densities by drag sampling along public hiking trails during the month of June. We quantified the infection prevalence of blacklegged nymphs at a total of five sites, and to aid in the design of future monitoring efforts, we determined whether infection levels differed significantly among the four study sites on the island of Nantucket. 

Despite a large body of previous research documenting tick-borne diseases on Nantucket, e.g., [20,23,34,35,36,37,38,39,40], quantitative surveillance of the abundance of infected blacklegged nymphs has not been reported. Moreover, few previous studies have documented the early establishment of lone star ticks as they spread to new locations along the coast of New England [11,41,42]. To our knowledge, this is the first published report of lone star establishment on Nantucket Island. 

A further consideration for choosing to study these islands is that small coastal islands are attractive sites for testing various types of wildlife interventions to prevent tick-borne diseases, e.g., [43,44], and baseline data are needed to gauge the efficacy of such efforts. Specifically, Buchthal et al. [45] proposed releasing white-footed mice that are genetically engineered to be resistant to *B. burgdorferi* on Nantucket and Martha’s Vineyard [45,46]. They plan to carry out preliminary field trials with genetically engineered mice on small islands in the region [45].

## 2. Materials and Methods

### 2.1. Study Sites

**Figure 1:**
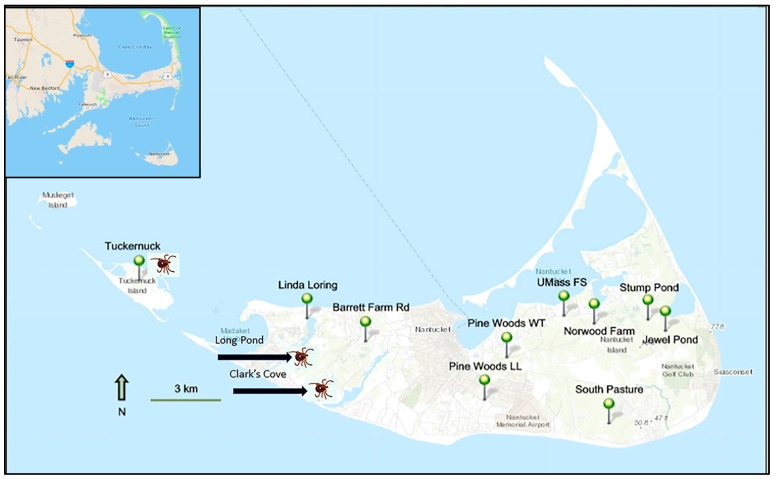
Map of nine study sites established on Nantucket Island and one on Tuckernuck Island in 2020. In 2021, two sites were added on Nantucket to document invading *Amblyomma americanum* (Long Pond and Clark’s Cove); tick symbol shows sites where *A. americanum* densities were measured. Inset map shows eastern Massachusetts.

Study sites were located on the islands of Nantucket (123 km^2^) and Tuckernuck (4.2 km^2^) in the town of Nantucket, Nantucket County, Massachusetts (Figure 1). Nantucket Island has a population of ~15,000 people year-round, increasing to ~60,000 residents and summer visitors in July and August [47], while Tuckernuck Island has <40 homes, all of which are seasonal. Deer densities on both islands are considered high, roughly estimated as >20 deer/km^2^ on Nantucket [48]. Geologically, these islands originated as part of a terminal moraine deposited ~15,000 years ago during the Wisconsin Glaciation [49]. Their topography includes upland glacial moraines, sandy outwash plains, freshwater wetlands, salt marshes, and barrier beaches. Soils are generally sandy, low in nutrients, and acidic, favoring plant communities dominated by oaks, pines, and ericaceous shrubs.

In 2020, we established 10 long-term study sites where it was possible to sample ticks along established trails in a variety of habitats, most of which were located on conservation preserves, and one of which was on Tuckernuck Island (Figure 1, Table 1, Supplementary Figure S1). These study sites were not intended to be representative of all tick habitats on the islands; rather, they were chosen as examples of common habitats that will serve as accessible sites for long-term monitoring of tick populations. Our choice of study sites across Nantucket included areas and habitats where lone star ticks are expected to spread in the future. On Tuckernuck, we did not include more than one site due to the island’s small size. In 2021, two additional sites were established on western Nantucket where lone star ticks had been observed. At all 12 study sites, property owners maintained the trails by annual brush-cutting as needed, and trails with grass were mowed once or twice during our fieldwork.

### 2.2. Drag Sampling

At each site, we sampled ticks along the edges of hiking trails that had leaf litter and/or low vegetation on and bordering the trail. The distances over which sampling was performed ranged from 0.40 km to 1.65 km per site (Table 1), depending on the local trail system and generally conforming to CDC recommendations to sample along a distance of at least 750 m for estimating tick densities [50]. Shrub thickets and a dense shrub understory in many wooded habitats precluded the use of replicated sampling within multiple plots, as used in other studies, e.g., [15,51,52]. Sampling along public trails allows our sites to be relocated easily by future investigators, including site managers and citizen scientists. 

To quantify the densities of blacklegged nymphs and lone star ticks, we used a common drag-sampling method that involved dragging a white cotton cloth over known distances [53,54,55]. Questing ticks cling to the drag cloth and are easily removed with silicone putty or a lint roller. Many questing ticks are likely to remain uncaptured after a single drag sweep [56] and a large fraction of the total population is not expected to be questing at any given time [57]. Nonetheless, sampling questing ticks with drag cloths is a widely accepted procedure for estimating relative densities [50]. 

A 1 m^2^ piece of white, rubberized flannel cloth with small lead weights sewn into the distal corners was dragged slowly over leaf litter and low vegetation along the edge of the trail and checked every 12 m [58]. *Ixodes* nymphs were removed from the drag cloth, counted, and those from five high-density sites were frozen for DNA analyses. If needed, a few extra sweeps were carried out on additional days to obtain a total of 300–400 nymphs for DNA analyses from each of the five high-density sites. We chose these target sample sizes to allow for site-specific confidence intervals of approximately ±5% infection prevalence each year. Lone star nymphs and adults captured from these same drag samples also were counted, as were clusters of at least 50 lone star larvae per 12 m drag sweep. 

Drag sampling was carried out between 3 and 30 June 2020, 1 and 26 June 2021, and 29 May and 27 June 2022, coinciding with the period of peak blacklegged nymphal abundance in coastal Massachusetts [20]. At any given site, the number of questing ticks collected during drag sampling can be highly variable from day to day [52,59]. Several steps were taken to standardize our sampling methods. First, the length of the trail at each site would be expected to cross multiple small clusters of questing nymphs, thereby representing average local densities and compensating for “hot spots” where deer may have rested [39,60]. We sampled each site on five days per year and alternated the order and times of day when each site was sampled. To reduce variation due to unfavorable conditions for questing, we used tick densities from the four days of sampling that had the greatest densities at each site to calculate average densities per km of trail per site. All fieldwork was conducted by the same person (A. Snow), and sampling was carried out when the vegetation was dry, typically before noon and after 1500 h to avoid mid-day heat on sunny days. Previous studies have shown that lone star ticks often quest during drier periods of the day than blacklegged ticks [61], so early afternoon sampling was included at study sites with lone stars. Adult *I. scapularis* were uncommon and, therefore, adult densities are not reported (no larvae were observed). Likewise, *Ixodes* nymphs were uncommon at Long Pond and Clark’s Cove, Nantucket, where lone star ticks were sampled in 2021 and 2022, so *Ixodes* densities are not reported for these two sites.

### 2.3. DNA Analyses for Species Identification and Pathogen Prevalence

*Ixodes* nymphs were transferred to vials and stored in a freezer for DNA analyses, which were performed on a subset of all sampled *Ixodes* nymphs from each of the five sites (Table 1). We analyzed DNA from >330 nymphs per site per year, for a total of 4212 nymphs. *Ixodes* nymphs were stored at −20 °C, sorted into individual tubes, and total nucleic acids were extracted from each tick using the Masterpure Complete DNA and RNA Purification Kit (Biosearch Technologies, WI, USA) following the manufacturer’s protocols. 

Tick species identification was determined using Taqman real-time PCR assays [62,63]; Supplementary Table S1). Briefly, a tick gene was used as an internal control for each sample, and differentiation of *I. scapularis* vs. *I. dentatus* was performed using assays specific to each tick species. A subset of nymphs that were collected in 2021 for DNA analyses were photographed under a Leica stereo-dissecting microscope to view morphological traits of DNA-confirmed samples of each species. For nymphs of both *Ixodes* species, we tested for the presence of six disease agents: *B. burgdorferi* (Bb), *B. miyamotoi* (B miya), *B. mayonii*, *Babesia microti* (Bm), *A. phagocytophilum* (Ap), and the *Ehrlichia-muris*-Like Agent (EMLA) using the methods in Xu et al. [62,63]. Probes and primers used for pathogen identification are listed in Supplementary Table S1. We did not identify which Ap variants were present in our Ap-positive samples (see Section 4). 

We report 95% confidence intervals to compare nymphal infection prevalence among sites, between islands, and between years [64]. For nymphs infected with two or more pathogens, we tested for positive or negative associations between three pairs of pathogens (Bb+Bm, Bb+Ap, Bm+Ap) using Chi-square tests. If a nymph had three pathogens, it was included in analyses with each of these pairs for association tests. Expected frequencies for each pair were calculated as the product of each pathogen’s overall frequency. We also used Chi-square tests to determine whether triple-infected nymphs occurred more often than expected based on each pathogen’s overall frequency. The density of infected nymphs (DIN) was calculated as the product of the density of nymphs (DON) and nymphal infection prevalence (NIP).

### 2.4. Vegetation Surveys

We surveyed plant communities at each site to record current conditions and provide a baseline for future researchers. Plant communities along the selected trails occurred in a complex mosaic due to microsite variation in soil moisture, land use history, and management practices. To characterize plant communities, we recorded the presence of common woody species within a radius of 1m on each side of the trail at ~15 m intervals. At each of these observation points, we also recorded the presence of woody species with branches extending over the trail, the presence of tall shrubs (>2 m high) immediately adjacent to the trail (also providing shade), and the presence of open grassland areas lacking shade. These data were used to estimate the frequencies of shaded trails, common woody species, and adjacent grassland areas at each site (Supplementary Table S2, Supplementary Figure S2). 

## 3. Results

### 3.1. Tick Species Other than I. scapularis and A. americanum

We did not encounter *Dermacentor variabilis* (dog ticks) at the study sites, although they have been observed on Nantucket in the past [65]. A few nymphs and adults of rabbit-specific *Haemaphysalis leporispalustris* were collected each year (data not shown). No other tick species were identified with the exception of *Ixodes dentatus*, as noted below.

At the five study sites for which nymphal *Ixodes* DNA was tested, a few nymphs were identified as *I. dentatus* rather than *I. scapularis* (Supplementary Table S3, Supplementary Figure S3). In 2020, 10% of *Ixodes* nymphs from Norwood Farm were *I. dentatus*, as were 6% of those from the UMass Field Station, while only a few were found at the other three sites. A similar pattern was seen in 2021, with 4% identified as *I. dentatus* at Norwood Farm and 10% at UMass Field Station, and very few, if any, collected at the other three sites. We detected *B. burgdorferi* (Bb), *Babesia microti* (Bm), and *A. phagocytophilum* (Ap) in a few of these *I. dentatus* nymphs (Supplementary Table S3). 

### 3.2. Densities of Ixodes Nymphs 

Because we did not attempt to confirm the species identification of all observed *Ixodes* nymphs using DNA markers, we refer to these nymphs simply as “*Ixodes*” when summarizing the drag-sampling results. We presume that the vast majority of *Ixodes* nymphs were *I. scapularis*, especially at Stump Pond, Jewel Pond, and Tuckernuck Island, where <1–2% were *I. dentatus* based on DNA analyses (Supplementary Table S3). We typically captured ~0–5 *Ixodes* nymphs along each 12m section of the sampled trails, or occasionally up to ~10 nymphs per section at sites with the highest densities. Therefore, our reported densities at a given site represent nymphs that were collected across many microsite locations along the length of the sampled trails, averaged across four days of sampling per year. 

**Figure 2:**
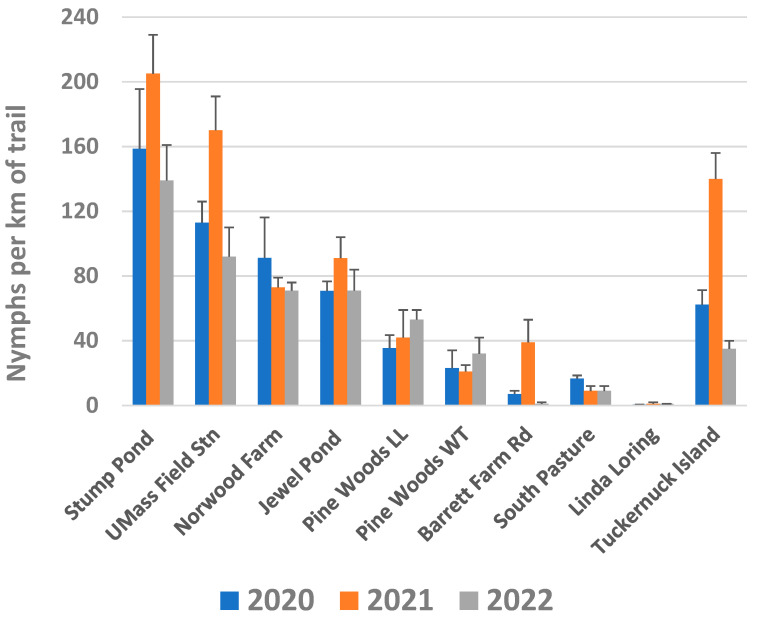
Density of *Ixodes* nymphs at 9 sites on Nantucket and one site on Tuckernuck Island in 2020–2021. Average of 4 sampling days per site per year (±1 SE).

Average nymphal densities were generally highest in 2021 and lower in 2020 and 2022, although not every site showed this pattern (Figure 2). Across the 10 study sites, we observed 37% more *Ixodes* nymphs/km in 2021 compared to 2020, and 9% fewer in 2022 compared to 2020. The relative nymphal density across the 10 sites was roughly consistent year-to-year, e.g., Stump Pond and UMass Field Station had the two highest densities each year while Linda Loring had the lowest (Figure 2). 

Sites with higher nymphal densities tended to have more shade than those with lower densities (Supplementary Table S2, Supplementary Figure S2). Scrub oak (*Quercus ilicifolia*) was common as a canopy species at Stump Pond, Norwood Farm, and Jewel Pond, while black oak (*Q. serotina*) and white oak (*Q. alba*) were dominant canopy tree species on Tuckernuck (Supplementary Table S2). Common shrub species at sites with high *Ixodes* densities included black huckleberry (*Gaylusaccia baccata*), viburnum (*Viburnum dentatum*), and beaked hazelnut (*Corylus cornuta*) in the understory of wooded areas, and bayberry (*Morella caroliniensis*) in sunnier, open microsites. Five study sites had lower nymphal densities, including the two Pine Woods sites, where much of the forest floor was carpeted with pine needles, and South Pasture, where scrub oak grows in a low, dense, nearly monospecific scrub thicket on dry, sandy soils. The lowest nymphal densities were found at the two grassland sites, Barrett Farm Road and Linda Loring, where no tree cover was present along the trails (Supplementary Figure S2). 

### 3.3. Densities of Lone Star Ticks 

**Figure 3:**
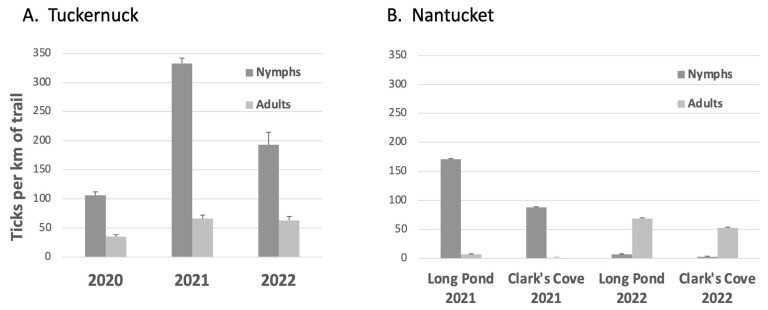
Average densities of *Amblyomma americanum* on (**A**) Tuckernuck Island (one site, 2020–2022), and (**B**) Nantucket (two sites, 2021, 2022). Averages (±1 SE) based on 4 days of sampling per site per year, except for 2021 on Nantucket with 3 days of sampling.

Lone star nymphs and adults were very abundant on Tuckernuck (Figure 3A). In 2021, lone star densities on Tuckernuck increased by 3.2-fold for nymphs and 2.3-fold for adults compared to 2020. Likewise, the total number of 12m sweeps yielding clusters of >50 lone star larvae increased from 6 in 2020 to 27 in 2021. In 2022, lone star nymphal densities decreased somewhat and were intermediate between densities observed in 2020 and 2021, while a total of 24 sweeps had clusters of >50 lone star larvae. 

Lone star ticks were extremely rare at the original nine study sites on Nantucket. At Linda Loring, we found 1 nymph in 2020, 43 nymphs and 2 adults in 2021, and 3 nymphs and 13 adults in 2022 (totals over 4 days of sampling). None were found at the UMass Field Station, Stump Pond, or Norwood Farm, and only 1–5 lone star nymphs or adults were found at the other five original study sites in 2020–2022. On 16 June 2021 and 15 June 2022, we also searched for lone star ticks by drag sampling along ~1 km at Coskata Woods in eastern Nantucket and did not find any.

In 2021, we established two new study sites on western Nantucket where locally abundant lone star ticks had been reported by colleagues (Table 1, Figure 1). At the Long Pond site, the wide, unshaded, mowed trail was bordered by tall shrubs and occasional trees (*Quercus ilicifolia*, *Prunus serotina*). The Clark’s Cove site consisted of a mowed trail through open grassland, adjacent to dense shrub thickets. At both sites, more nymphs than adults were observed in 2021, and more adults than nymphs in 2022 (Figure 3B). 

### 3.4. Infection Prevalence in Ixodes scapularis

**Figure 4:**
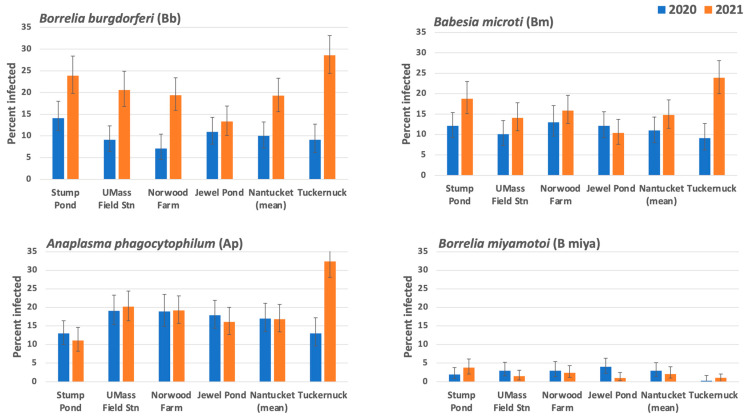
*Ixodes scapularis* nymphal infection prevalence for four sites on Nantucket Island and one site on Tuckernuck Island in 2020 vs. 2021. Shown with 95% CI; see Table 2 for sample sizes. Mean infection prevalence across four sites on Nantucket is shown with 95% CI based on mean sample sizes.

None of the 4071 *I. scapularis* nymphs tested were positive for EMLA (*Ehrlichia-muris*-Like Agent) or *Borrelia mayonii*, so these pathogens will not be considered further. *Borrelia miyamotoi* (B miya) infected <5% of nymphs at all five sites in both 2020 and 2021 (Figure 4). *Borrelia burgdorferi* (Bb), *Babesia microti* (Bm), and *A. phagocytophilum* (Ap) were common at all sites in both years. We used 95% CI to identify significant differences in the infection prevalence for Bb, Bm, Ap, and B miya across sites and years (Figure 4, N = 330–458).

#### 3.4.1. Comparisons among Sites on Nantucket

For Bb and Bm, infection levels were generally similar across sites within years (Figure 4). Bb prevalence increased significantly from 2020 to 2021 at three of the four sites. Ap infection levels were similar across sites, although somewhat lower at Stump Pond, and were consistent between years. At all sites on Nantucket, coinfections with Bb and Bm were significantly more common than expected due to chance (Table 2; *p* < 0.05 or *p* < 0.01, Chi-square tests). These two pathogens co-occurred in 3–4% of nymphs at each site in 2020 and 6–9% of nymphs in 2021. Coinfections with Ap were rare and were no more or less common than expected by chance. 

#### 3.4.2. Comparisons between Nantucket and Tuckernuck

For ease of presentation, we averaged the infection prevalence for the four sites on Nantucket for each pathogen in each year (Figure 4). Average infection prevalence on Nantucket was 10% vs. 19% (2020–2021) for *Borrelia burgdorferi*, 11% vs. 15% (2020–2021) for *Babesia microti*, and 17% (both years) for *Anaplasma phagocytophilum*. Frequencies of all three pathogens increased dramatically on Tuckernuck in 2021 compared to 2020, from 9% to 29% for Bb, 9% to 24% for Bm, and 13% to 32% for Ap. Based on non-overlapping 95% CI levels, the increased prevalence observed in 2021 was significant for Bb on Nantucket and for all three pathogens on Tuckernuck (Figure 4). Densities of infected nymphs (DIN) also were greater in 2021 compared to 2020 due to increases in both abundance and infection prevalence (Table 3).

Coinfections were found in an average of 7% of nymphs on Nantucket and 6% on Tuckernuck in 2020 vs. 11% on Nantucket and 25% on Tuckernuck in 2021 (Table 2). In 2020 and 2021, respectively, an average of 37% and 41% of nymphs that were Bb-positive nymphs on Nantucket were coinfected with Bm, while 52% and 54% of Bb-positive nymphs on Tuckernuck were coinfected with Bm.

## 4. Discussion

### 4.1. Densities of Ixodes Nymphs

Mean densities of *Ixodes* nymphs (presumably *I. scapularis*; see Section 3) were greatest at sites with the most tree canopy, shade, and shrub cover, and lowest in open grasslands, consistent with many previous studies in the northeastern USA [66]. Four of the five high-density study sites had abundant oaks, which provide acorn mast for wildlife as well as shade and leaf litter for ticks. At the UMass Field Station, we found high densities of nymphs despite a dearth of mature tree cover, but much of the trail was shaded by tall shrubs. 

Previous studies also are consistent with our results showing greater nymphal densities at four sites dominated by a mix of oaks and other deciduous species compared to two sites dominated by conifers [66,67,68]. One low-density site on Nantucket, South Pasture, was dominated by low-growing scrub oak, which offered shade and shelter from wind, but had dry soil and low plant diversity (Supplementary Table S2). Two grassland sites with very little shade, Linda Loring and Barrett Farm Road, had the fewest nymphs/km. Our findings from the grassland sites are consistent with common public health recommendations for keeping lawns and walkways mowed and clear of brush and leaf litter to minimize exposure to blacklegged nymphs [4,69]. 

During the three years of this study, nymphal densities peaked in 2021 compared to 2020 and 2022 at several sites. Many previous studies have shown year-to-year variation in tick densities. Stafford et al. [30] sampled woodland habitats at the same 8–10 residential sites in southern Connecticut from 1989–1997 and reported a 4.7-fold variation in nymphal densities over the years. In Dutchess County, NY, Ostfeld et al. [70] reported 2–3-fold more nymphs in forested sites in 1994 compared to the previous two years. These authors and others have tested for correlations between blacklegged tick densities and factors such as acorn production, the abundance of bloodmeal host species, and extremes of temperature or precipitation [15,71]. A cold, dry winter in the previous year may result in greater nymphal mortality [27,72], while extremely dry and hot weather in summer can cause nymphs to spend less time questing [73]. Ostfeld et al. [74] reported increases in nymphal densities two years after masting in oak-dominated forests, which they attributed to population increases in white-footed mice and eastern chipmunks. 

### 4.2. Densities of Lone Star Ticks

The expanding range of lone star ticks in New England now includes populations in coastal New York, Connecticut, Rhode Island, Massachusetts, and Maine [10,11,22,42]. Compared to blacklegged ticks, lone star ticks lay more eggs per female (~5000 vs. 3000), are more tolerant of desiccation, have better nymphal survival, are more attracted to CO_2_ emitted by hosts, quest for bloodmeals in more habitats, move greater distances, move more quickly, and do not rely on small mammals for bloodmeals during their immature stages, instead feeding primarily on deer [11,61,66,75].

Lone star abundance can far exceed that of blacklegged ticks [7,68], as we observed each year on Tuckernuck Island. Lone star ticks have been a noticeable nuisance on Tuckernuck since at least 2015, yet they are still rare on Nantucket. The sharp contrast between these two islands in lone star densities is puzzling, given their close proximity (<4 km apart), similar habitats, and similar weather conditions. Lone star larvae and nymphs can disperse via birds [76], but they do not seem to spread evenly as they disperse to new areas. Similar to Tuckernuck, isolated populations of lone star ticks have been found on Manresa Island, CT, and Prudence Island, RI [8,22]. Lone star populations at Long Pond and Clark’s Cove on Nantucket exhibited year-to-year fluctuations in the relative densities of nymphs vs. adults during the sampling period, but they are now relatively common at these two sites. Clusters of lone star larvae have been found nearby [77], confirming that females are reproducing. We expect that eventually lone star ticks will become more widely established across Nantucket, but it is not possible to predict how long this could take. 

Lone star ticks do not carry *B. burgdorferi,* and their infection levels for causal agents of tularemia, ehrlichiosis, heartland virus disease, and infection with *Borrelia lonestari* appear to be low in Massachusetts [11,78]. Only 2% of lone star ticks from Massachusetts that were submitted to the TickReport public testing program in 2015–2021 were positive for a tested pathogen (N = 464, [78]). In contrast, Williams et al. [44] tested 100 lone star adults and 104 nymphs from Manresa Island, CT, and found disease agents for ehrlichiosis in 47% of adults and 9% of nymphs. A major health concern regarding lone star ticks is acquiring the alpha-gal allergy to red meat [13].

### 4.3. Infection Prevalence in Ixodes scapularis Nymphs

Nymphal infection levels are related to the local abundance of pathogen reservoir species that are available to larvae. Only four terrestrial mammal species have been observed on Tuckernuck: Deer, white-footed mouse, eastern cottontail rabbit (*Sylvilagus floridianus*), and meadow vole (*Microtus pennsylvanicus*). These species also occur on Nantucket, along with the northern short-tailed shrew (*Blarina brevicada*), eastern gray squirrel (*Sciurus carolinensis*), and several other species, but not the eastern chipmunk (*Tamias striatus* [46]. Bb and Bm can be transmitted to *I. scapularis* by white-footed mice, shrews, and other species [36,79,80,81,82,83], but see [39]. For *A. phagocytophilum*, variant-ha (Ap-ha) is transmitted by white-footed mice and causes human anaplasmosis, while variant-1 (Ap-v1) is associated with deer and other ruminants and is not known to be pathogenic in humans [32,84,85,86,87]. 

Many studies report nymphal infection prevalence (NIP) for common pathogens of *I. scapularis*, but smaller sample sizes and the different scales over which sampling occurred can make it challenging to compare other findings with those reported here. With this caveat in mind, we note that average infection levels for Bb across the Nantucket sites were generally comparable to the range of values reported in other northeastern states, e.g., [32,88,89]. We found that Bb NIP nearly doubled at three sites on Nantucket and tripled on Tuckernuck in 2021 (Figure 4). Year-to-year variation in Bb NIP also has been found in previous studies [29,30,90,91]. In our study, the average Bm NIP across sites was 11% in 2020 and 15% in 2021 (Figure 4), similar to several previous studies [80,91,92], while values of only ~3–5% Bm NIP were found in others [28,32,88,89,93]. We found that Ap NIP averaged 17% on Nantucket in both years. However, Ap NIP on Tuckernuck jumped from 13% in 2020 to 32% in 2021, similar to increases seen for Bb and Bm on this island (Figure 4). Other studies report values of Ap NIP below 10%, e.g., [32,92].

Several publications from the northeastern USA reported *B. burgdorferi* (Bb) as the most common pathogen carried by blacklegged nymphs, usually occurring much more frequently than *Babesia microti* (Bm) or *A. phagocytophilum* (Ap) [26,28,32,88,89,92,93]. In contrast, our data show that the prevalence of Bm and Ap were generally similar to or greater than the prevalence of Bb within years (Figure 4; also seen by Jordan et al. [91]. To some extent, this difference could be related to how long the pathogens have been common in different regions. Babesiosis and Lyme disease have been endemic on Nantucket for at least 40 years [20,34], unlike other areas of New England and Canada where increases in the range and prevalence of Bb have preceded the more recent spread of Bm [1,36,94,95]. Anaplasmosis was recorded on Nantucket in 1994 [37] and is still considered to be an emerging disease in much of the northeastern USA [3]. 

Although we found comparable nymphal infection levels for Bb, Bm, and Ap in this study, we note that Lyme disease is typically much more common than babesiosis and anaplasmosis where these three disease agents co-occur [1,3]. To explore this pattern further for residents of Nantucket, we queried the Massachusetts Virtual Epidemiological Network [96] and found that cases of Lyme disease were reported 4.5× more often than babesiosis and 10× more often than anaplasmosis in 2017–2021. Many factors could be responsible for lower numbers of reported cases of babesiosis and anaplasmosis, but we suspect that a portion of the Ap-infected nymphs on Nantucket may have the Ap-v1 variant. Further research focusing on the frequency of the human infective Ap-ha variant vs. non-infective Ap-v1 in both field-collected ticks and passive surveillance from tick-testing services is needed to better understand this disease risk [84,87,97,98]. 

### 4.4. Coinfections in Ixodes scapularis Nymphs

Nymphs that are infected with more than one pathogen pose an elevated health risk for people who acquire more than one disease from them [99]. On Nantucket, 3–7% of nymphs were coinfected with Bb+Bm in 2020 and 2021, respectively, and 37–41% of nymphs that were Bb-positive also tested positive for Bm, posing a greater health risk than either pathogen alone. Coinfection with these two pathogens was even more common on Tuckernuck. Many previous authors also report that coinfections with Bb+Bm occurred more frequently than expected based on the overall prevalence of each pathogen individually [28,92] and references therein, presumably because larvae fed on reservoir hosts that were coinfected with both pathogens. In addition, laboratory experiments suggest that coinfection with Bb+Bm in white-footed mice appears to facilitate the transmission of Bm to larvae of blacklegged ticks [94]. 

Coinfections involving Ap are not expected to be more common than random expectations unless the Ap variant commonly co-occurs with Bb or Bm in a reservoir host. Surprisingly, 7% of the nymphs from Tuckernuck had triple coinfections (Bb+Bm+Ap) in 2021, and another 9% were coinfected with either Bb+Ap or Bm+Ap (Table 2). Because Ap was strongly associated with Bb and Bm at this site, we suspect that many nymphs had the human-infective Ap-ha variant acquired from white-footed mice. Consistent with expectations about different reservoir hosts for each Ap variant, Edwards et al. [93] reported a positive association for Bb+Ap-ha in coinfected nymphs from eastern Pennsylvania but not for Bb+Ap-v1, which was more common overall.

## 5. Conclusions

This research was designed to serve as an example of how a small field crew can be deployed to monitor ticks and tick-borne disease agents at the scale of a local community. Drag sampling along public trails is a simple procedure that can be undertaken by citizen scientists with a minimum level of training, but analyzing samples for pathogen prevalence requires substantial expertise and funding. Unlike most previous studies, we sampled 300–400 blacklegged nymphs per site to be able to report the percent infected by each pathogen with relatively narrow 95% confidence intervals (±5%) for each site. Because we did not find significant differences among the Nantucket sites, future efforts could save time and funding by analyzing an equal but smaller number of samples from several different sites, for a total of ~300–400 nymphs. Sampling could be carried out every few years to inform public health officials about which pathogens are most common and to check for newly emerging disease agents and tick species.

Efforts to quantify and compare tick abundances among different studies are inherently challenging due to the use of different sampling methods, such as timed sampling vs. sampling over a given distance, as well as day-to-day and year-to-year variation in local tick densities. To help mitigate this problem, we recommend sampling at least 4 times during peak nymphal abundance and reporting tick densities per distance sampled (per km or m^2^), over a distance of at least 500–750 m at each study site, building on similar recommendations in the literature [50,100].

For blacklegged nymphs, we recorded density increases that co-occurred with increases in Bb NIP at several of our study sites, thereby amplifying the risk of exposure to tick-borne pathogens (Table 3). Abundances of blacklegged nymphs were greatest in 2021 on both islands, in synchrony with lone star abundances on Tuckernuck Island, suggesting that a common but unknown set of conditions may have favored both tick species in 2021. We also document the establishment of lone star ticks on the western portion of Nantucket Island. The Asian longhorned tick (*Haemaphysalis longicornis*), which has recently spread to eastern Long Island, NY, and Block Island, RI [42,101,102], is an exotic tick species that was not observed in our study and bears watching in the future. This species was found on Nantucket in 2023 [103]. Newly established pathogens also could become established in the future. For example, we did not detect *Ehrlichia muris eauclairensis* in nymphs of *I. scapularis* at our study sites, but this pathogen was recently found in Massachusetts [104].

In summary, our research characterizes current conditions and provides a baseline for further monitoring of ticks and tick-borne disease agents in the town of Nantucket, MA. By sampling tick densities and determining the prevalence of tick-borne disease agents at permanent study sites, we obtained data that can be compared with other studies where similar methods are employed. Due to the abundance of ticks and tick-borne pathogens on Nantucket, continued education and vigilance are needed to warn people about the risk of infections and coinfections involving Bb, Bm, Ap, and, to a lesser extent, B miya, as well as the risk of acquiring the alpha-gal red meat allergy from lone star ticks. 

## Figures and Tables

**Table 1:** Characteristics and locations of study sites on Nantucket and Tuckernuck islands. Asterisks indicate sites where infection prevalence was determined for blacklegged nymphs. See Figure 1 for map of study sites.

Site Name	Year Sampled	Vegetation	Trail Distance (km)	Lat/Long at 0 km	Property Owner
Tuckernuck Island * (1 site)	2020–2022	Mature oak woods, high mesic shrubs	1.65	41°18′13.827″ N, 70°15′23.997″ W	Tuckernuck Land Trust, private property
Nantucket Island (11 sites)					
Stump Pond *	2020–2022	Mixed woods, high mesic shrubs	0.96	41°17′13.184″ N, 69°59′43.139″ W	Nantucket Islands Land Bank, Nantucket Conservation Foundation
UMass Field Station *	2020–2022	Successional shrubs and grass	0.54	41°17′33.409″ N, 69°59′43.139″ W	Nantucket Conservation Foundation
Norwood Farm *	2020–2022	Mixed woods, high mesic shrubs	0.94	41°17′25.567″ N, 70°1′26.811″ W	Nantucket Conservation Foundation
Jewel Pond *	2020–2022	Mixed woods and scrub oak	0.94	41°17′19.029″ N, 69°59′27.947″ W	Mass Audubon
Pine Woods—Lovers Lane	2020–2022	Mixed conifer forest, shrub border	0.50	41°15′38.205″ N, 70°4′47.223″ W	Commonwealth of MA
Pine Woods—Water Tower	2020–2022	Open/disturbed pitch pine woods	0.64	41°16′37.853″ N, 70°4′15.578″ W	Commonwealth of MA
South Pasture	2020–2022	Low and medium-height scrub oak	1.10	41°15′6.790″ N, 70°0′42.788″ W	Nantucket Conservation Foundation
Barrett Farm Rd	2020–2022	Grassland adjacent to high mesic shrubs	0.78	41°16′51.682″ N, 70°8′42.164″ W	Nantucket Islands Land Bank
Linda Loring	2020–2022	Grassland with low heath shrubs	1.50	41°17′32.323″ N, 70°10′11.418″ W	Linda Loring Nature Foundation
Long Pond (lone star tick site)	2021, 2022	Grassy path through high mesic shrubs	1.40	41°16′19.079″ N, 70°10′53.758″ W	Nantucket Islands Land Bank
Clark’s Cove (lone star tick site)	2021, 2022	Grassland with low heath shrubs	0.40	41°15′54.623″ N, 70°9′51.466″ W	Nantucket Conservation Foundation

**Table 2:** Percent of all *Ixodes scapularis* nymphs with coinfections at each site on Nantucket and Tuckernuck islands in 2020 and 2021. Sample sizes for percentages are in parentheses. Pathogen abbreviations are Bb (*Borrelia burgdorferi*), Bm (*Babesia microti*), and Ap (*Anaplasma phagocytophilum*). Coinfections with *Borrelia miyamotoi* are not included as a separate category due to small sample sizes. Where * *p* < 0.05 or ** *p* < 0.01 in Chi-Square tests, coinfections were more common than expected.

Island	Site	Year	Total Nymphs	Percent Infected	Co-Infected	Bb+Bm	Bb+Ap	Bm+Ap	Bb+Bm+Ap
Nantucket	All sites	2020	1614	35	7	3 *	1	1	0.6
				(562)	(108)	(51)	(19)	(18)	(9)
	Stump Pond	2020	448	31	8	3 *	2	2	1
				(141)	(35)	(14)	(8)	(10)	(3)
	UMass Field Stn	2020	398	34	6	3 *	1	1	1
				(135)	(25)	(13)	(3)	(4)	(2)
	Norwood Farm	2020	338	38	5	2 *	1	1	1
				(128)	(17)	(8)	(3)	(2)	(3)
	Jewel Pond	2020	430	37	7	4 *	1	0.5	0.2
				(158)	(31)	(16)	(5)	(2)	(1)
Nantucket	All sites	2021	1683	41	11	7 **	2	1	1
				(685)	(187)	(116)	(29)	(17)	(15)
	Stump Pond	2021	398	42	13	9 **	0	2	2
				(169)	(52)	(34)	(1)	(7)	(6)
	UMass Field Stn	2021	412	42	13	7 **	3	1	1
				(175)	(52)	(27)	(14)	(4)	(5)
	Norwood Farm	2021	458	46	10	6 **	2	1	0.4
				(210)	(48)	(29)	(8)	(6)	(2)
	Jewel Pond	2021	415	32	8	6 **	1	0	0.5
				(131)	(35)	(26)	(6)	(0)	(2)
Tuckernuck	Tuckernuck	2020	330	25	6	5 *	1	1	0
				(82)	(21)	(15)	(2)	(4)	(0)
	Tuckernuck	2021	444	54	25	8 **	7 *	2	7 **
				(241)	(109)	(37)	(29)	(10)	(31)

**Table 3:** Density of infected *Ixodes* nymphs (DIN) per km of trail on each island in 2020 and 2021. DIN is the product of the density of nymphs (DON) and nymphal infection prevalence (NIP). For Nantucket, DON and DIN are based on the average of four study sites for which NIP was determined. Sample sizes as in Figure 2 (DON) and Table 2 (NIP). Pathogen abbreviations as in Figure 4.

Location	Year	DON	Bb DIN	Bm DIN	Ap DIN	B miya DIN
Nantucket	2020	108	11	12	18	3
	2021	138	27	20	23	3
Tuckernuck	2020	62	6	6	8	0
	2021	140	40	33	45	2

## Data Availability

Data are available from the corresponding author (A.S.) upon request.

## Supplementary Materials

The following supporting information can be downloaded at: https://www.mdpi.com/article/10.3390/insects14070628/s1. Figure S1. Maps and habitat photos of the sampled trails at each study site. Figure S2. Frequency of tree canopy, high shrubs, and no shade along trails sampled at each site, based on 33–111 sample points per site. Figure S3. Representative photos of *Ixodes dentatus* vs. *Ixodes scapularis* showing morphological similarity between species. Table S1. Primers and probes used in DNA analyses for species ID and pathogens. Table S2. A. Frequency of tree canopy species with branches over the trail at 10 study sites. B. Frequency of common woody species and vines within 1m of the trail at 10 study sites. Table S3. Frequency and infection status of *Ixodes dentatus* nymphs collected at study sites on Nantucket and Tuckernuck islands in 2020 and 2021.
