# Supplementary material for: Tick Densities and Infection Prevalence on Coastal Islands in Massachusetts, USA: Establishing a Baseline
Source: Insects. 2023 Jul 12;14(7):628. doi: 10.3390/insects14070628 (PMC10380421; doi:10.3390/insects14070628)
Supplement: Supplementary file 1 [file insects-14-00628-s001.zip › insects-2461304-supplementary - proof-v1/Suppl Table 1. Primers and probes used in DNA analyses..pdf]

Table S1. Primers and probes used in DNA analyses for tick species identification and pathogens.

Target	Primer or Probe Names	Primer or Probe Sequences
Tick internal control	HardTick_18S_F1	AATGATTTAGTGAGGTCTTCGGACC
	HardTick_18S_R1	TGTTACGACTTTTACTTCCTCTAAATGATC
	HardTick_18S_P1_FAM	TCATCTTTCCAACACACCGGCGCAACC
Ixodes scapularis	I_scapularis_ITS_F1	TTTCTTTGAGCAAATGCACGAGTG
	I_scapularis_ITS_R1	GATTTTCCACAAACGGTATCCATCG
	I_scapularis_ITS_HEX_P1	CGCTTAACCAGTCCTCCTCCTCCTACGAGTT
Ixodes dentatus	I_dentatus_F2	TCTTGGCGTGGATGTTGTTCG
	I_dentatus_R2	GAAGGGTGTGCAAATCAACGC
	I_dentatus_FAM_P2	AGAAAGCTTCTGGGATGGAGTGACGGA
Borrelia genus	Borrelia_Genus_F3	GCTTCGCTTGTAGATGAGTCTGC
	Borrelia_Genus_R3	CCGTAGGAGTCTGGACCGTATC
	Borrelia_Genus_P3_705	TCCAGTGTGACCGTTCACCCTCTCAGGC
Borrelia burgdorferi Sensu Lato	BBSL_LNA_F1	ATAGGTCTAATATTAGCCTTAATAGCAT
	BBSL_LNA_R1	AGATCGTACTTGCCGTCTT
	BBSL_LNA_P1_FAM	aagc+Aaa+Atgtt+Agc+Agccttga
Borrelia miyamotoi	BMI_F1	AGACATAGTTCTAACAAAGGACAATATTCC
	BMI_R1	AGTTCAGTTAGTGTGAAGTCAGTAGC
	miyamotoi-GX-P_HEX	TGCACGACCCAGAAATTGACACAACCACAA
Borrelia mayonii	B_mayonii_ospC_F	TTGGTTGCATCCATAGATGAACTTG
	B_mayonii_ospC_R	CGGTTGATATTGTATAGATTCCTGATAGC
	B_mayonii_ospC_P_Cy5	CCATTCTTACCTGCTTCATTCCCCAACCCATTATTTT
Babesia microti	B-microti-F	GATTTGGAACCTGGCACCATG
	B-microti-R	AATGACCCTTAGCCCAATTATTTCC
	B-microti_P_FAM	ATCTGGCCCATACGGTGAATTGTTTCGC
Anaplasma	Anaplasma_F	ATGGAAGGTAGTGTTGGTTATGGTATT
	Anaplasma_R	TTGGTCTTGAAGCGCTCGTA

	Anaplasma_P_HEX	TGGTGCCAGGGTTGAGCTTGAGATTG
Ehrlichia muris-Like Agent	EMLA_F	TACCTAATTCTTCTCAAGAGATTCAGTTG
	EMLA_R	ATGATGATACTGCGAACAACTATAAGAG
	EMLA_P_Cy5	ATATTGATAAAAGAGTCAGTGTTGATCCGTATGAGTTAGGGTT
