# Supplementary material for: Tick Densities and Infection Prevalence on Coastal Islands in Massachusetts, USA: Establishing a Baseline
Source: Insects. 2023 Jul 12;14(7):628. doi: 10.3390/insects14070628 (PMC10380421; doi:10.3390/insects14070628)
Supplement: Supplementary file 1 [file insects-14-00628-s001.zip › insects-2461304-supplementary - proof-v1/Suppl Table 3. - Ixodes dentatus frequency and NIP.pdf]

Supplementary Table S3. Frequency and infection status of *Ixodes dentatus* nymphs collected at study sites on Nantucket and Tuckernuck islands in 2020 and 2021. All other *Ixodes* nymphs were *I. scapularis*. Pathogen abbreviations are Bb (*Borrelia burgdorferi*), Bm (*Babesia microti*), Ap (*Anaplasma phagocytophilum*).

Location	Year	Total *Ixodes* nymphs collected	Number *I. dentatus*	Percent *I. dentatus*	Number *I. dentatus* with at least one pathogen	Number *I. dentatus* with Bb only	Number *I. dentatus* with Ap only	Number *I. dentatus* with Bm only	Number *I. dentatus* with Bb + Bm
**Nantucket**									
**Stump Pond**	2020	454	6	1.3	0	0	0	0	0
**Stump Pond**	2021	399	1	0.3	0	0	0	0	0
**UMass Field Stn**	2020	423	25	**5.9**	1	1	0	0	0
**UMass Field Stn**	2021	457	45	**9.9**	11	9	1	0	1
**Norwood Farm**	2020	374	36	**9.6**	3	2	0	1	0
**Norwood Farm**	2021	477	19	**4**	3	1	0	2	0
**Jewel Pond**	2020	437	7	1.6	0	0	0	0	0
**Jewel Pond**	2021	415	0	0	0	0	0	0	0
**Tuckernuck**	2020	332	2	0.3	2	2	0	0	0
**Tuckernuck**	2021	444	0	0	0	0	0	0	0
