# Supplementary material for: Tick Densities and Infection Prevalence on Coastal Islands in Massachusetts, USA: Establishing a Baseline
Source: Insects. 2023 Jul 12;14(7):628. doi: 10.3390/insects14070628 (PMC10380421; doi:10.3390/insects14070628)

Figure S2. Frequency of tree canopy, high shrubs, and no shade along trails sampled at each site, based on 33-111 sample points per site. Order of presentation for each site as in Figure 2 showing tick densities. Frequencies of tree and shrub species at each site are listed in Supplemental Table 1.

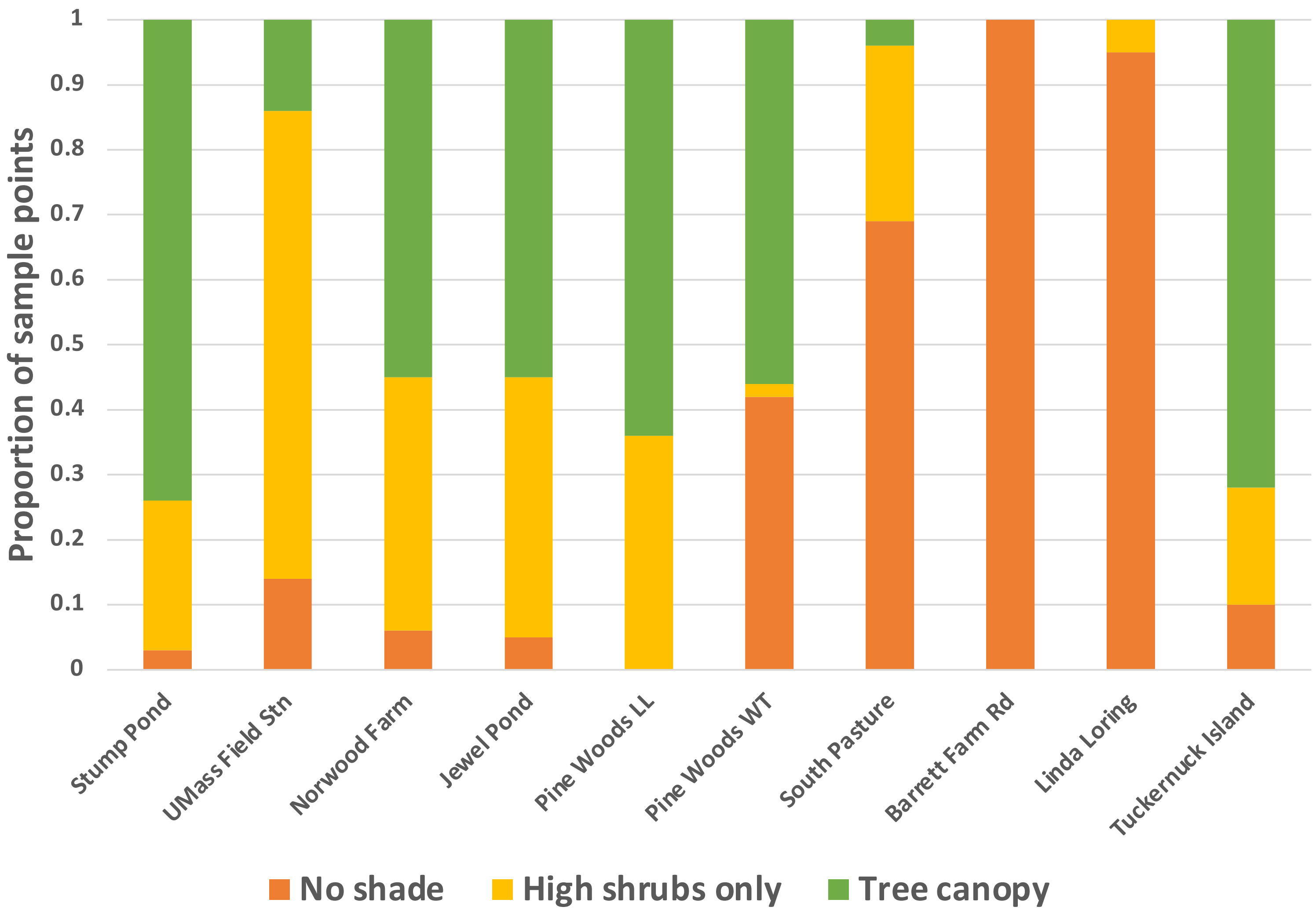

Supplement: Supplementary file 1 [file insects-14-00628-s001.zip › insects-2461304-supplementary - proof-v1/Supplementary Figure S2 - Freq of shade at each site.pdf]
