# Supplementary material for: Tick Densities and Infection Prevalence on Coastal Islands in Massachusetts, USA: Establishing a Baseline
Source: Insects. 2023 Jul 12;14(7):628. doi: 10.3390/insects14070628 (PMC10380421; doi:10.3390/insects14070628)

Figure S3. Representative photos of *Ixodes dentatus vs. Ixodes scapularis* nymphs showing morphological similarity between species.

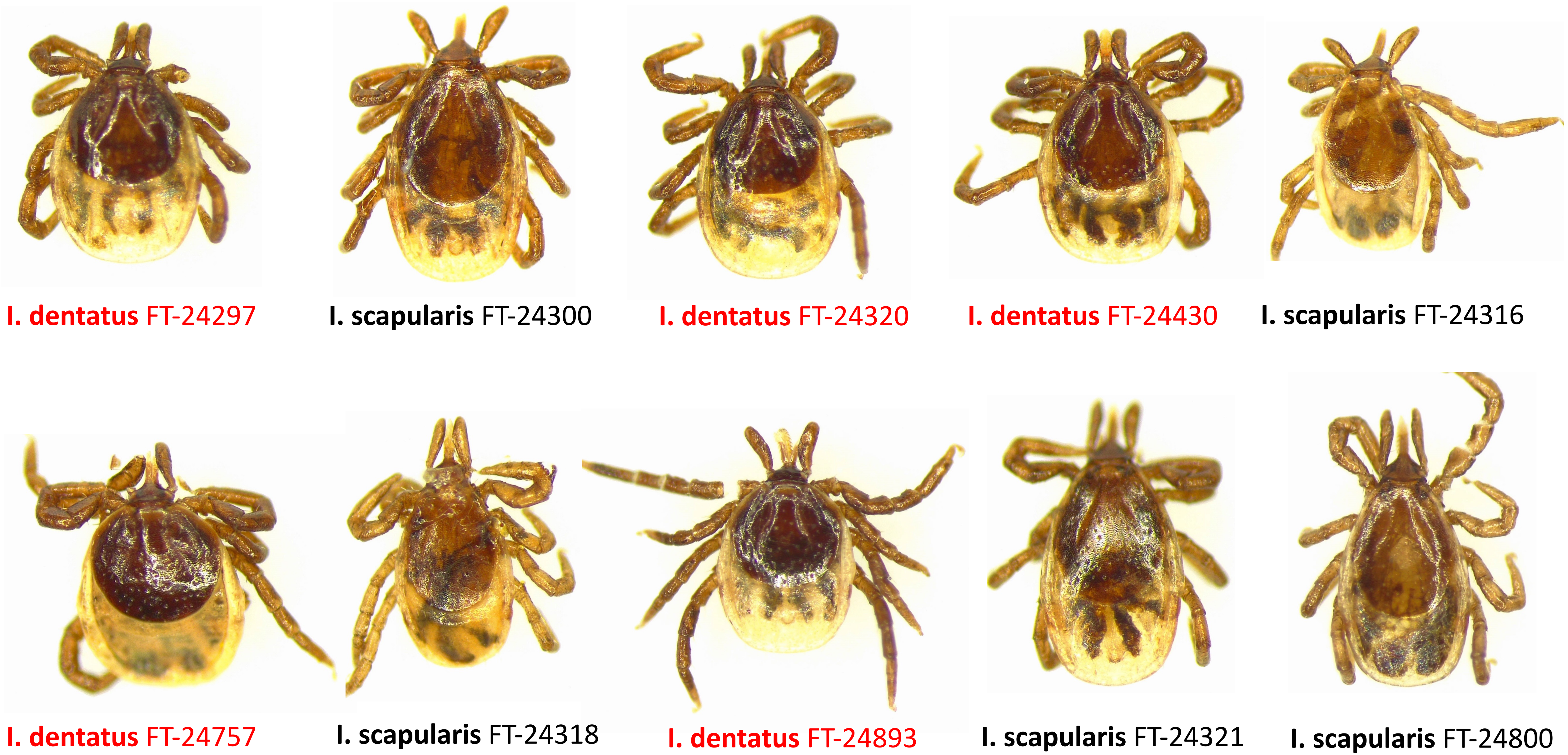

Supplement: Supplementary file 1 [file insects-14-00628-s001.zip › insects-2461304-supplementary - proof-v1/Supplementary Figure S3 - Photos of Ixodes scapularis vs. I. dentatus nymphs..pdf]
