# Supplementary material for: Tick Densities and Infection Prevalence on Coastal Islands in Massachusetts, USA: Establishing a Baseline
Source: Insects. 2023 Jul 12;14(7):628. doi: 10.3390/insects14070628 (PMC10380421; doi:10.3390/insects14070628)
Supplement: Supplementary file 1 [file insects-14-00628-s001.zip › Suppl Table 2. Woody plant frequencies at study sites.pdf]

Supplementary Table 2A. Frequency of tree canopy species with branches over the trail at 10 study sites.
Percent occurrence is shown (N = number of sample points); blank cells represent zero percent. When >1 tree species is present at a sample point, percent occurrence values for each site may sum to >100%. Sample points were spaced at regular intervals along each trail.

	**Sentinel Sites:**	Stump Pond	UMass Field Station	Nor-wood Farm	Jewel Pond	Tucker-nuck	Pine Woods LL	Pine Woods WT	South Pasture	Barrett Farm Road	Linda Loring
	N:	(60)	(35)	(49)	(57)	(102)	(33)	(45)	(75)	(55)	(110)
**Species:**	**Common names:**										
Quercus spp.	all oak spp.	63		37	47	70		2	4		
Quercus ilicifolia	scrub oak	63		27	47	5		2	3		
Prunus serotina	black cherry	8	6	2	5	8	6				
Acer rubrum	red maple	2									
Quercus alba	white oak			10		33					
Quercus velutina	black oak					44					
Sassafras albidum	sassafras					8					
Juniperus virginiana	red cedar		9								
Nyssa sylvatica	blackgum			6							
Pinus rigida	pitch pine			6			15	51			
Pinus strobus	white pine						39				

Populus grandidentata	aspen							7			
Amelanchier spp.	shadbush										
Crataegus monogyna	hawthorn										

Supplementary Table 2B. Frequency of common woody species and vines within 1m of the trail at 10 study sites.

Percent occurrence is shown if ≥10%; blank cells represent <10%. Sample sizes as in Table S1A, above. Small oak saplings are included in the data set.

**Species**	**Common name**	Stump Pond	UMass Field Station	Nor-wood Farm	Jewel Pond	Tucker-nuck	Lost Farm	Pine Woods LL	Pine Woods WT	South Pasture	Barrett Farm Road	Linda Loring
Quercus ilicifolia	scrub oak	92		92	91	19	34	97	91	100		11
Gaylusaccia baccata	black huckleberry	83		29	93	79			20	76		45
Corylus cornuta	beaked hazelnut	38		41	65	44		52	11			
Viburnum dentatum	viburnum	35	29	55	65	75	68	70	11			
Clethra alnifolia	sweet pepperbush	33		39								
Smilax rotundifolia	greenbriar	23	14	31								
Pteridium aquilinum	bracken fern	15			14							

Morella caroliniensis	bayberry	13	83	53	19	12	17	61	62	45		43
Vaccinium spp.	unidentified vaccinium	12			14							
Vitus labrusca	fox grape	10	71			11	21				55	
Toxicoden-dron radicans	poison ivy		23				64	39	21			20
Parthenocissus cinquefolia	Virginia creeper		20	14		20	70	24				
Prunus serotina	black cherry		11	16		17	75	39	11	28		15
(many species)	grass/forbs		54	31	21	30	23	36	22	16	100	78
Ligustrum spp.	privet		14									
Juniperus virginiana	juniper, red cedar		20									

Lonicera mackii	bush honeysuckle		40				21					
Amelanchier spp.	shadbush, serviceberry			12								
Quercus alba	white oak			12		23						
Sassafras albidum	sassafras			10		15						
Pinus rigida	pitch pine						11		33			
Arctostaphylos uva-ursi	red bearberry								51	47		
