# Supplementary material for: Tick Densities and Infection Prevalence on Coastal Islands in Massachusetts, USA: Establishing a Baseline
Source: Insects. 2023 Jul 12;14(7):628. doi: 10.3390/insects14070628 (PMC10380421; doi:10.3390/insects14070628)

Figure S1. Maps and habitat photos for the 12 study sites.

Latitude and longitude are listed for the beginning of each trail.				
**Site**	Property owner	Trail length (km)	Latitude	Longitude
**Stump Pond**	Nantucket Land Bank, Nantucket Conservation Foundation	0.96	41° 17' 13.184" N	69° 59' 43.139" W
**UMass Field Station**	Nantucket Conservation Foundation	0.54	41° 17' 33.409" N	70° 2' 20.123" W
**Norwood Farm**	Nantucket Conservation Foundation	0.94	47° 17' 25.567" N	70° 1' 26.811" W
**Jewel Pond**	Mass Audubon Society	0.94	41° 17' 19.029" N	69° 59' 27.947" W
**Tuckernuck Island**	Tuckernuck Land Trust, private property	1.65	41° 18' 13.827" N	70° 15' 23.997" W
**Pine Woods – Lover's Lane**	Commonwealth of MA	0.50	41° 15' 38.205 N	70° 4' 47.223" W
**Pine Woods – Water Tower**	Commonwealth of MA	0.64	41° 16' 37.853" N	70° 4' 15.578" W
**South Pasture**	Nantucket Conservation Foundation	1.10	41° 15' 6.790" N	70° 0' 42.788" W
**Barrett Farm Road**	Nantucket Land Bank	0.78	41° 16' 51.682" N	70° 8' 42.164" W
**Linda Loring**	Linda Loring Foundation	1.50	41° 17' 32.323" N	70° 10' 11.418" W
**Long Pond (lone star ticks)**	Nantucket Land Bank	1.4	41° 15' 54.623" N	70° 9' 51.466" W
**Clark's Cove (lone star ticks)**	Nantucket Conservation Foundation	0.4	41° 16' 19.079" N	70° 10' 53.758" W

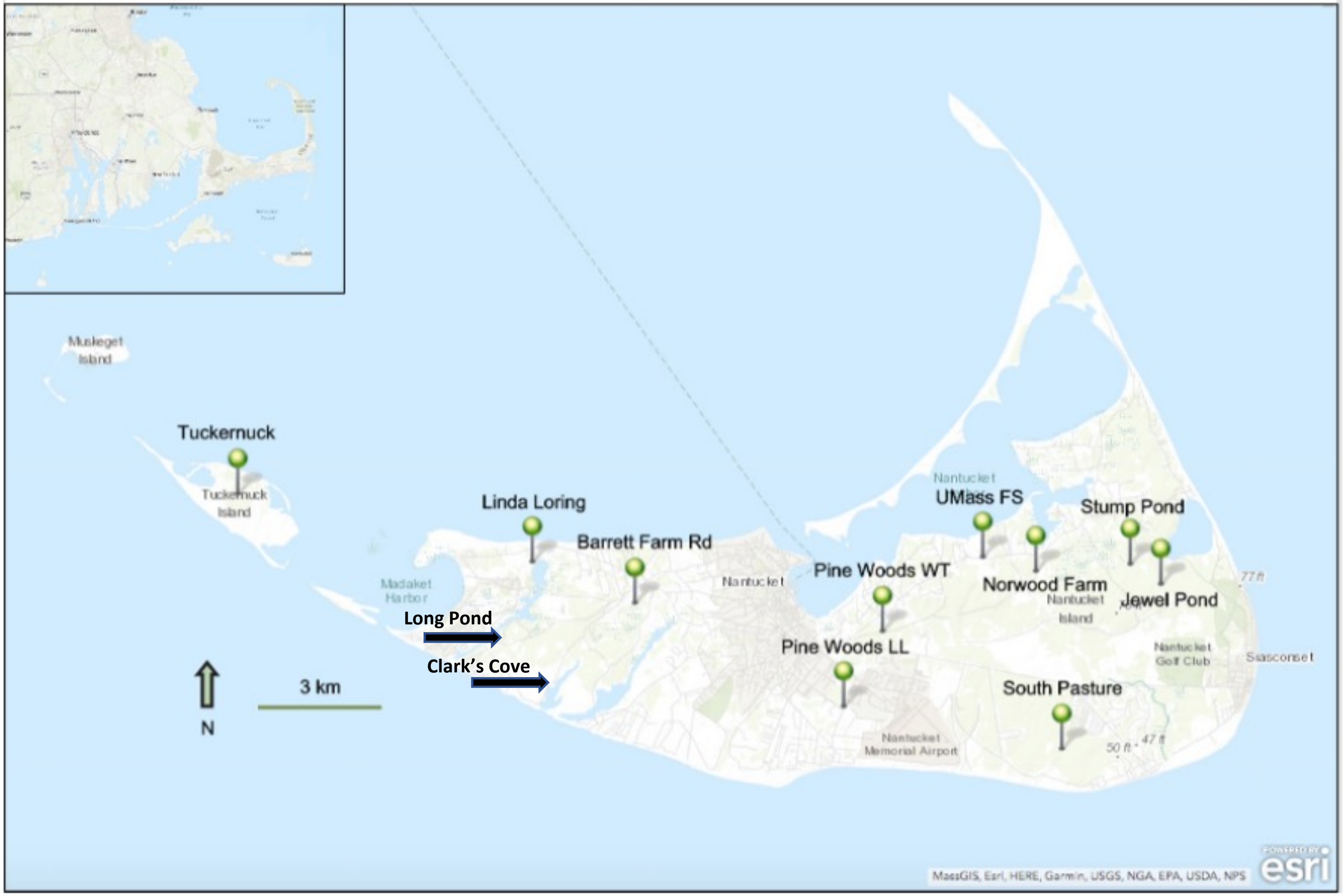

Muskeget Island
Tuckernuck
Tuckernuck Island
Linda Loring
Barrett Farm Rd
Madaket Harbor
Long Pond
Clark's Cove
Nantucket
Pine Woods WT
Pine Woods LL
UMass FS
Stump Pond
Norwood Farm
Jewel Pond
Nantucket Island
Nantucket Golf Club
Siasconset
South Pasture
Nantucket Memorial Airport
77 ft
50 ft
47 ft
3 km
N
MassGIS, Esri, HERE, Garmin, USGS, NGA, EPA, USDA, NPS

Tuckernuck Site (trail is divided into nearby sections)

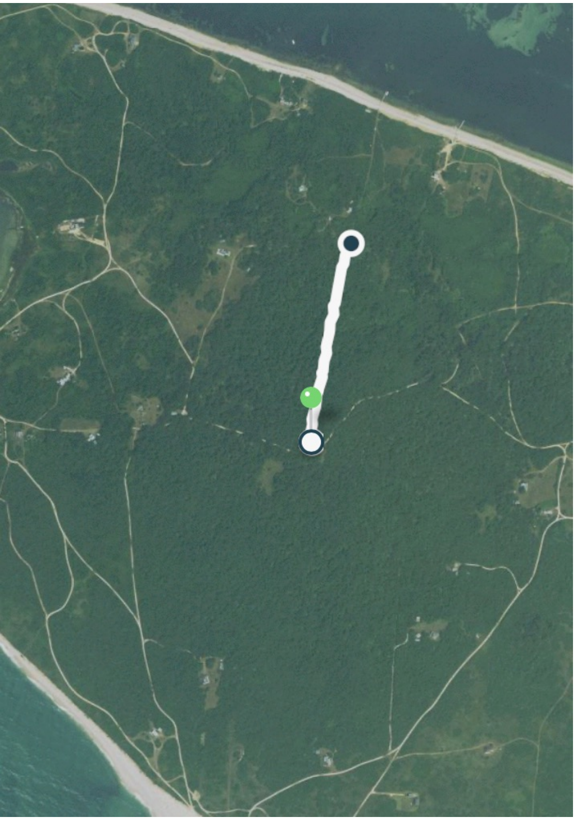

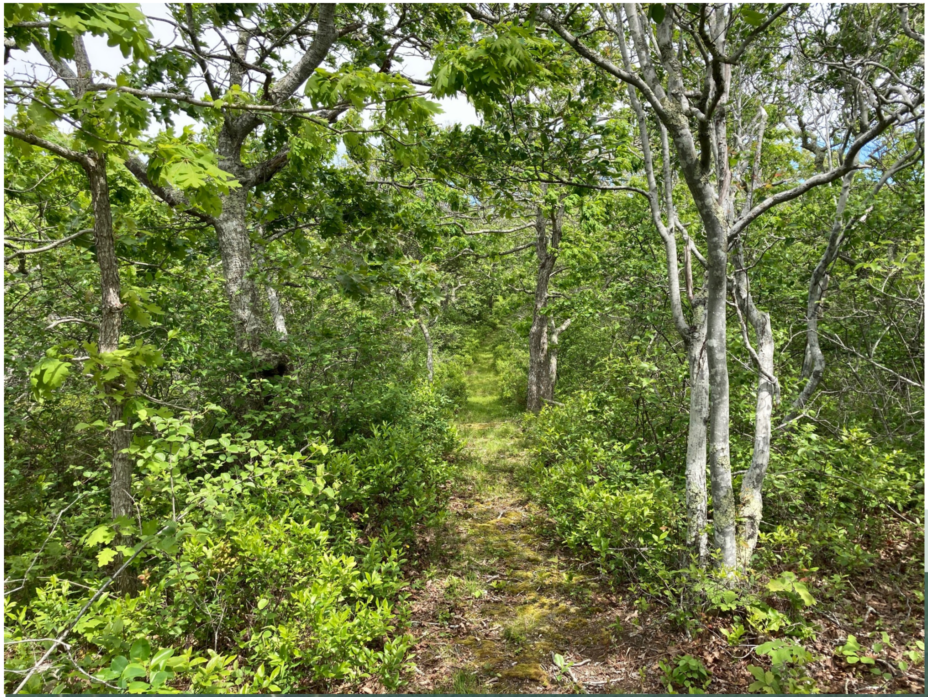

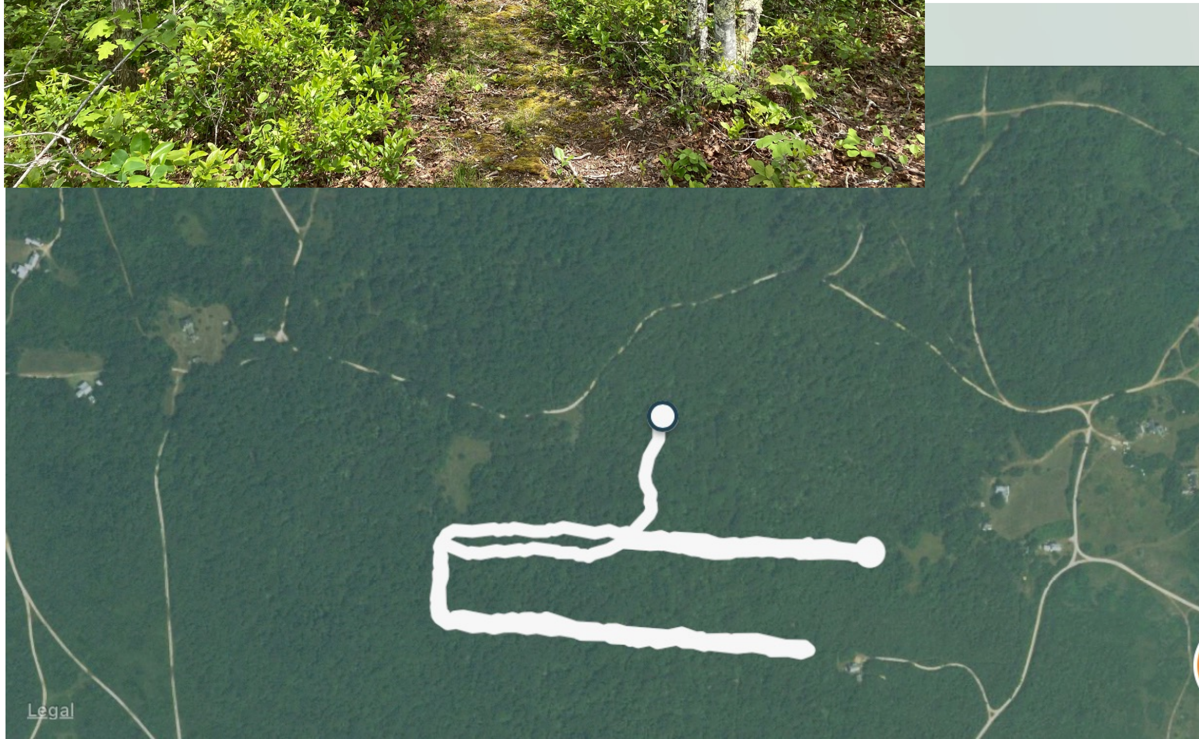

Stump Pond Site – access is via 345 Polpis Rd.

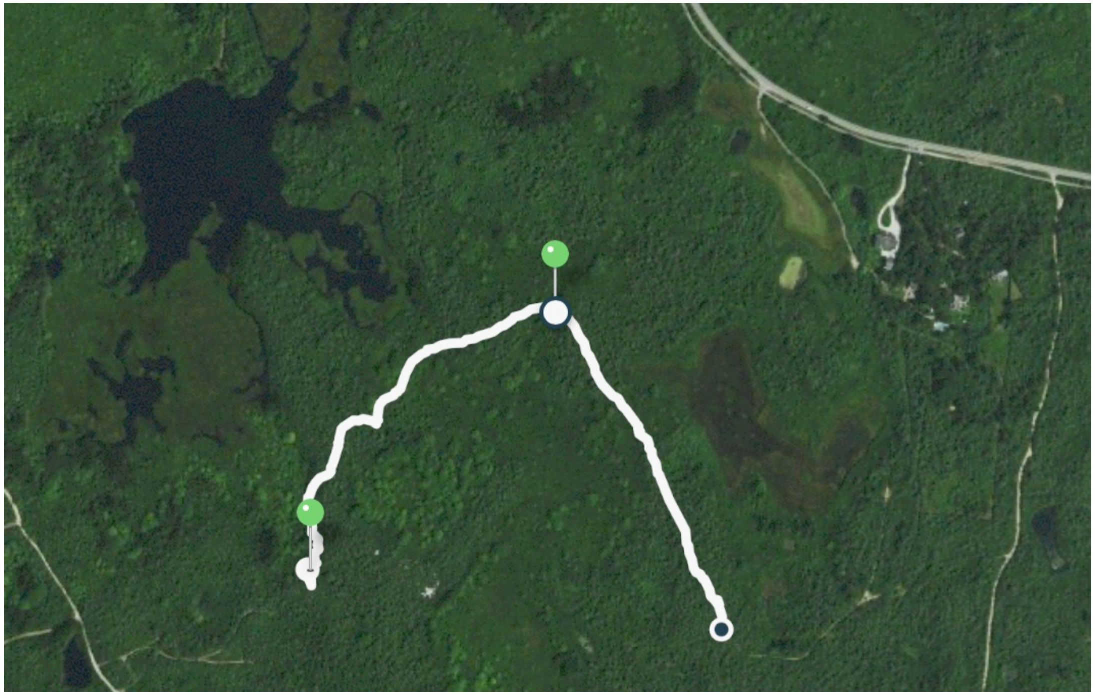

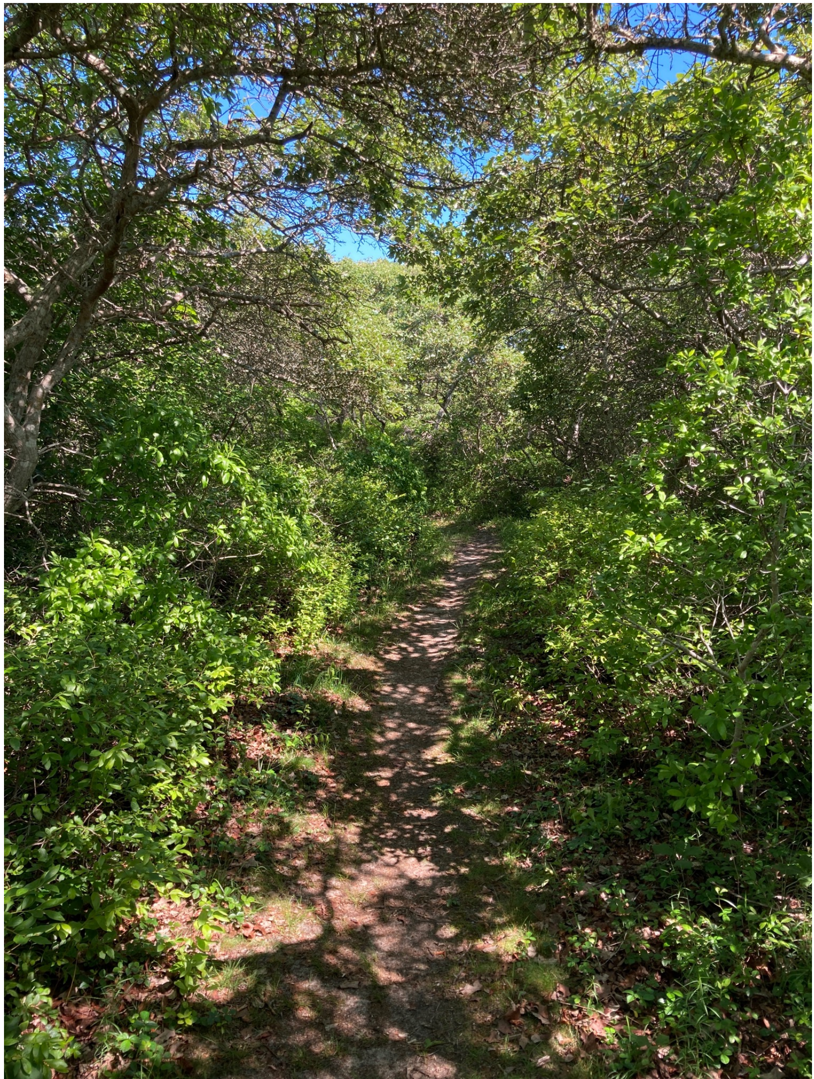

UMass Field Station Site – access at 180 Polpis Rd.

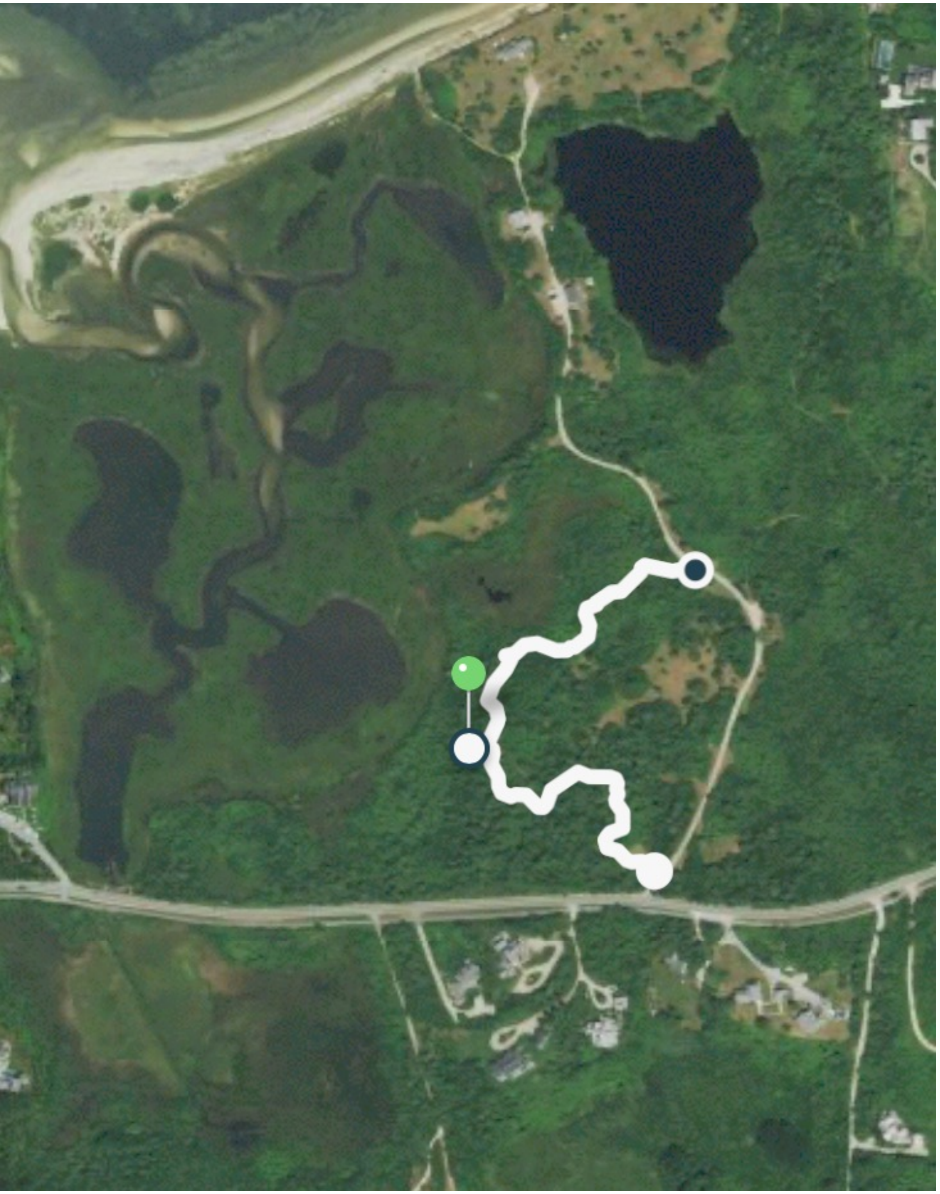

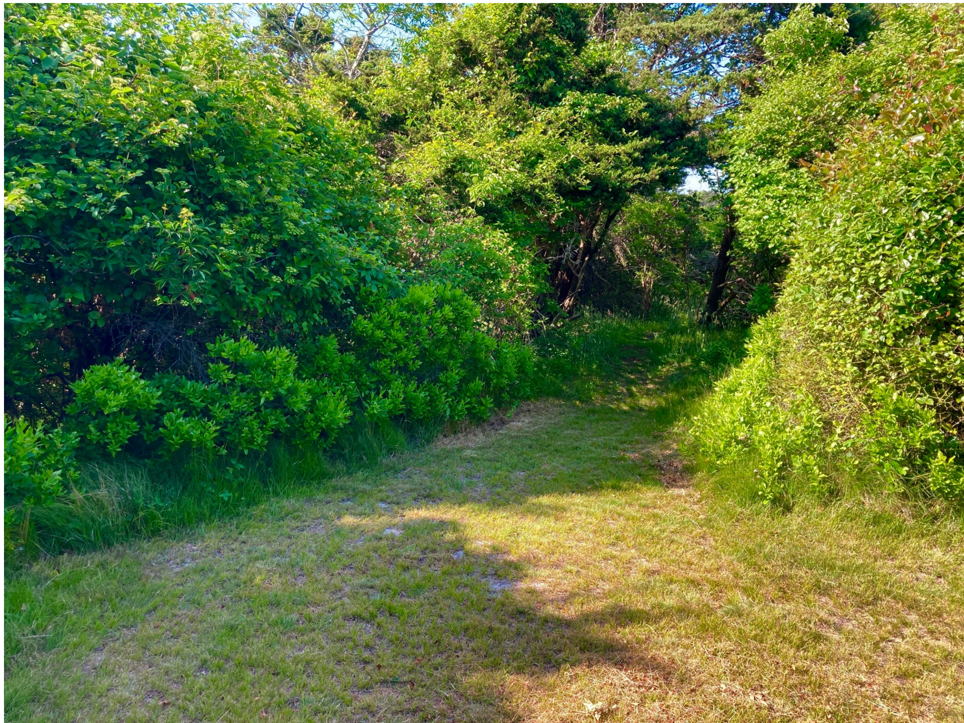

Norwood Farm Site – Old Polpis Rd.

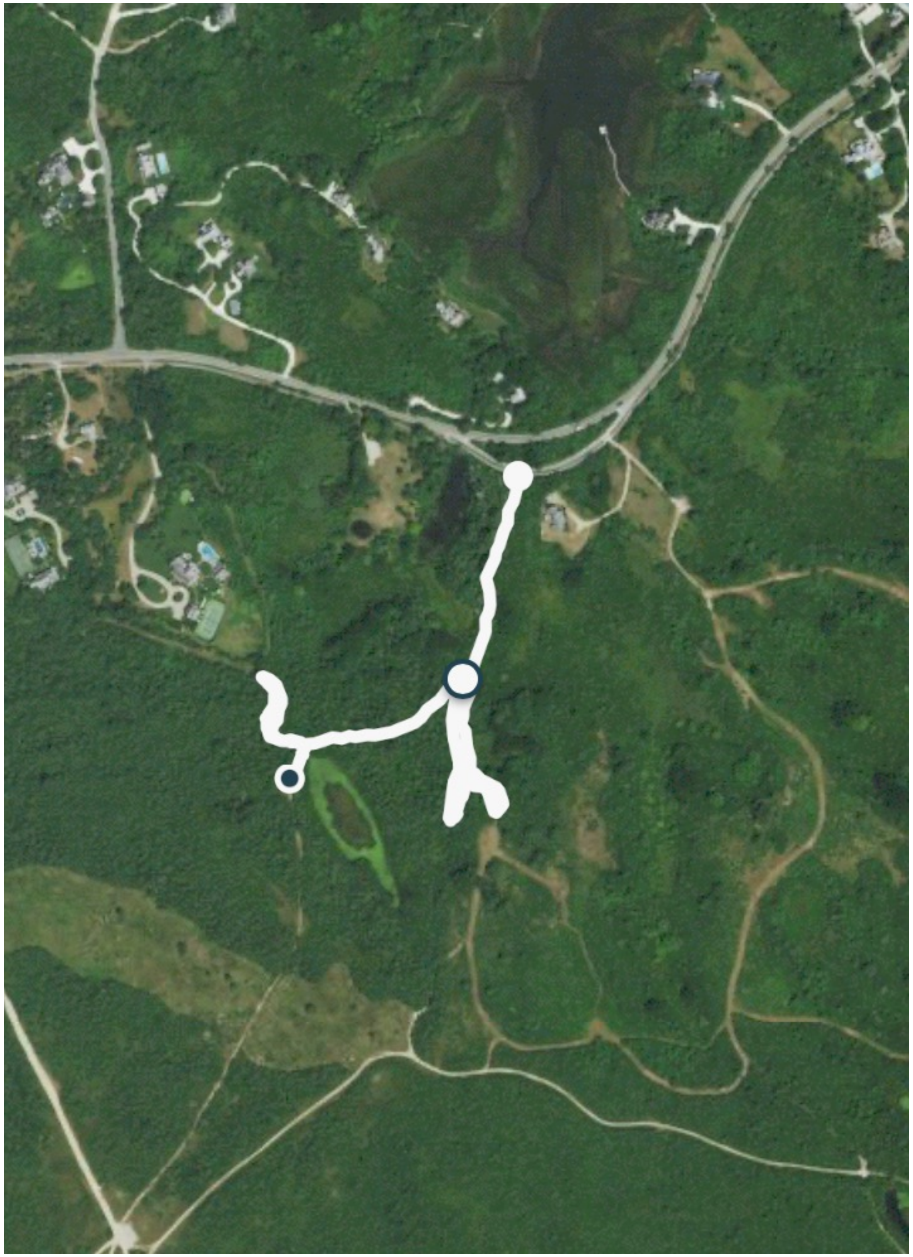

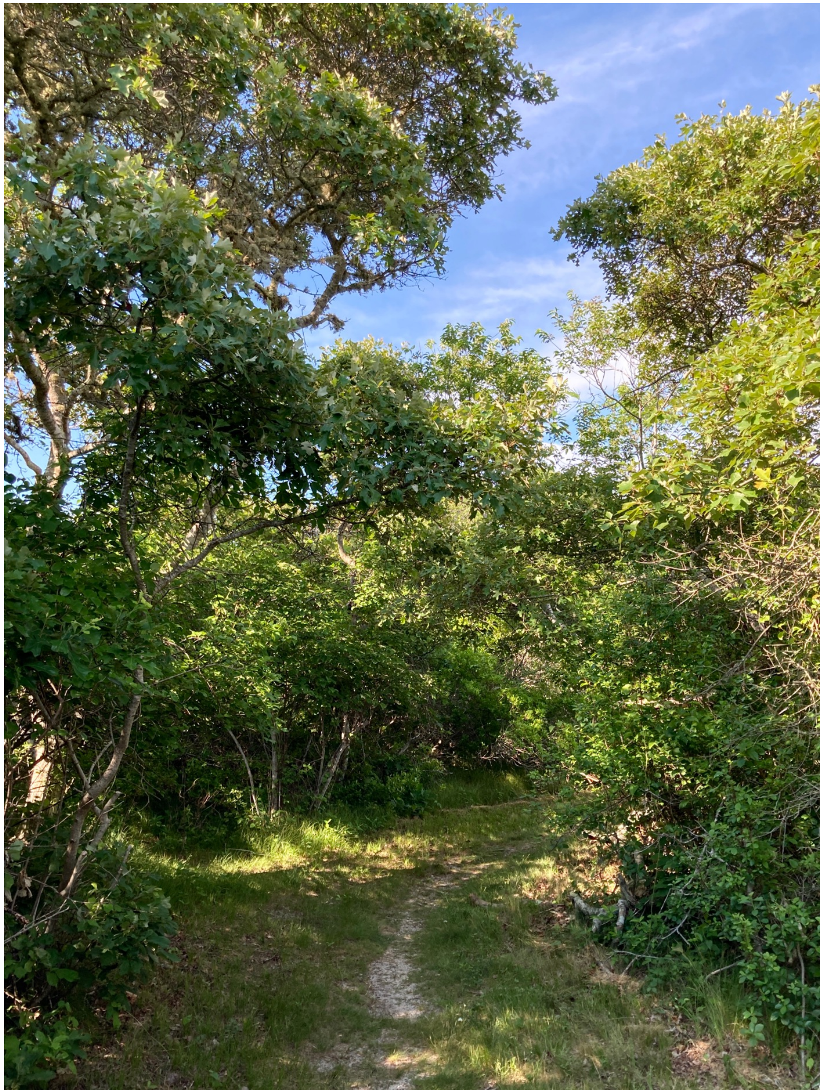

Jewel Pond Site – access via 345 Polpis Rd.

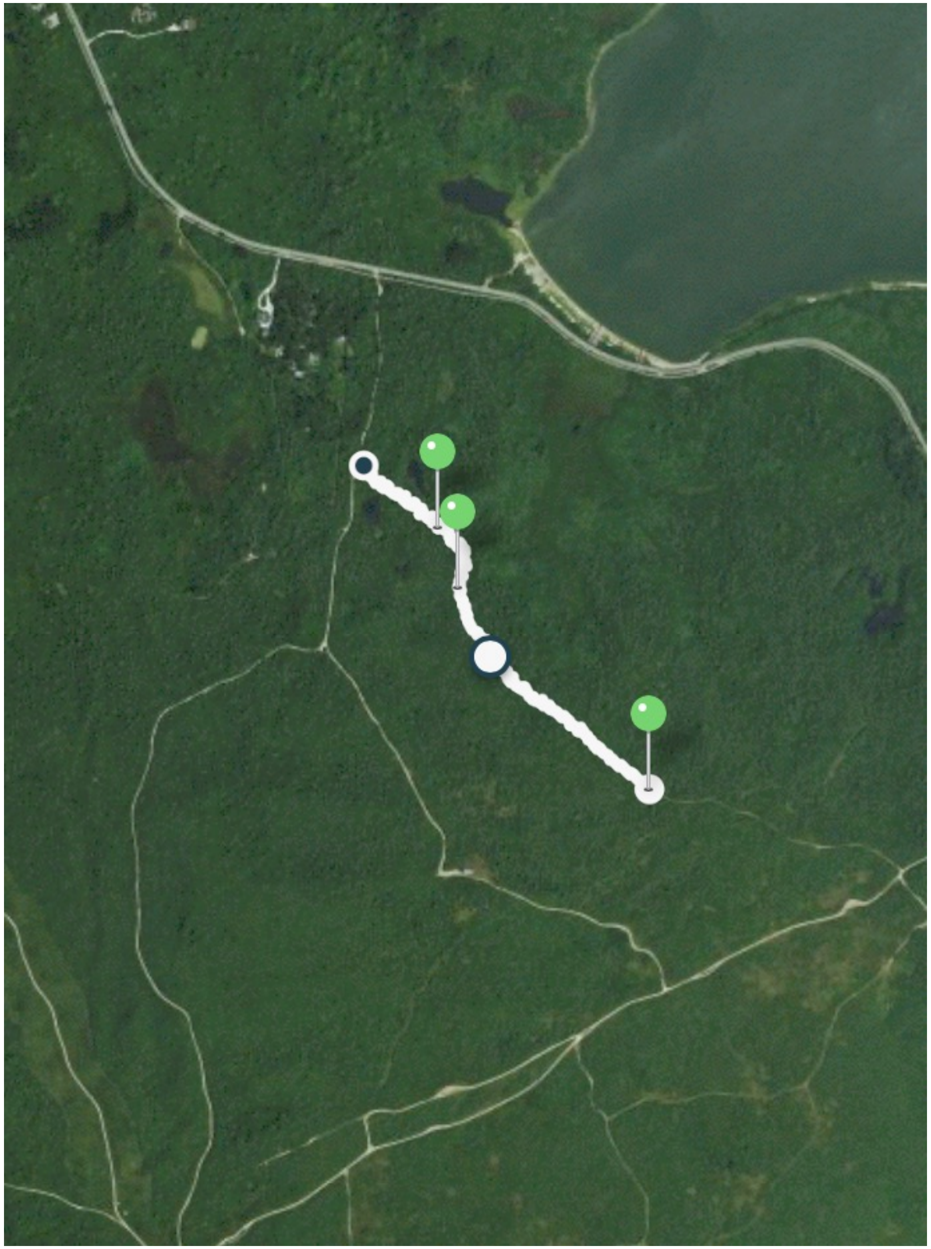

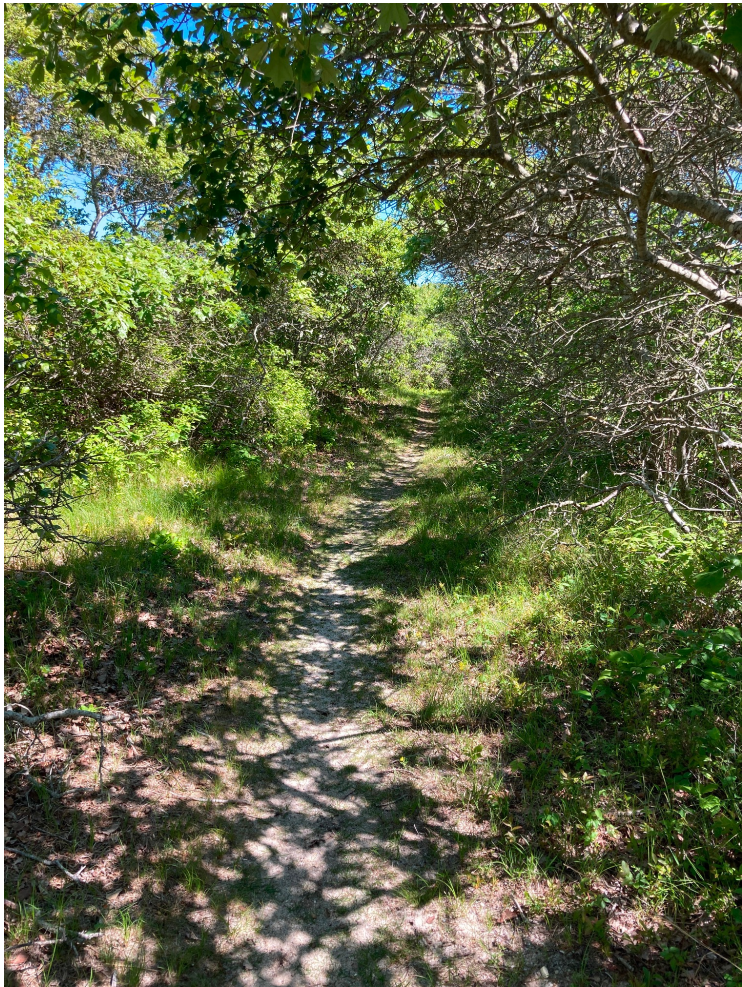

Pine Woods, Lovers Lane Site – corner of Rugged Rd and Lovers Lane.

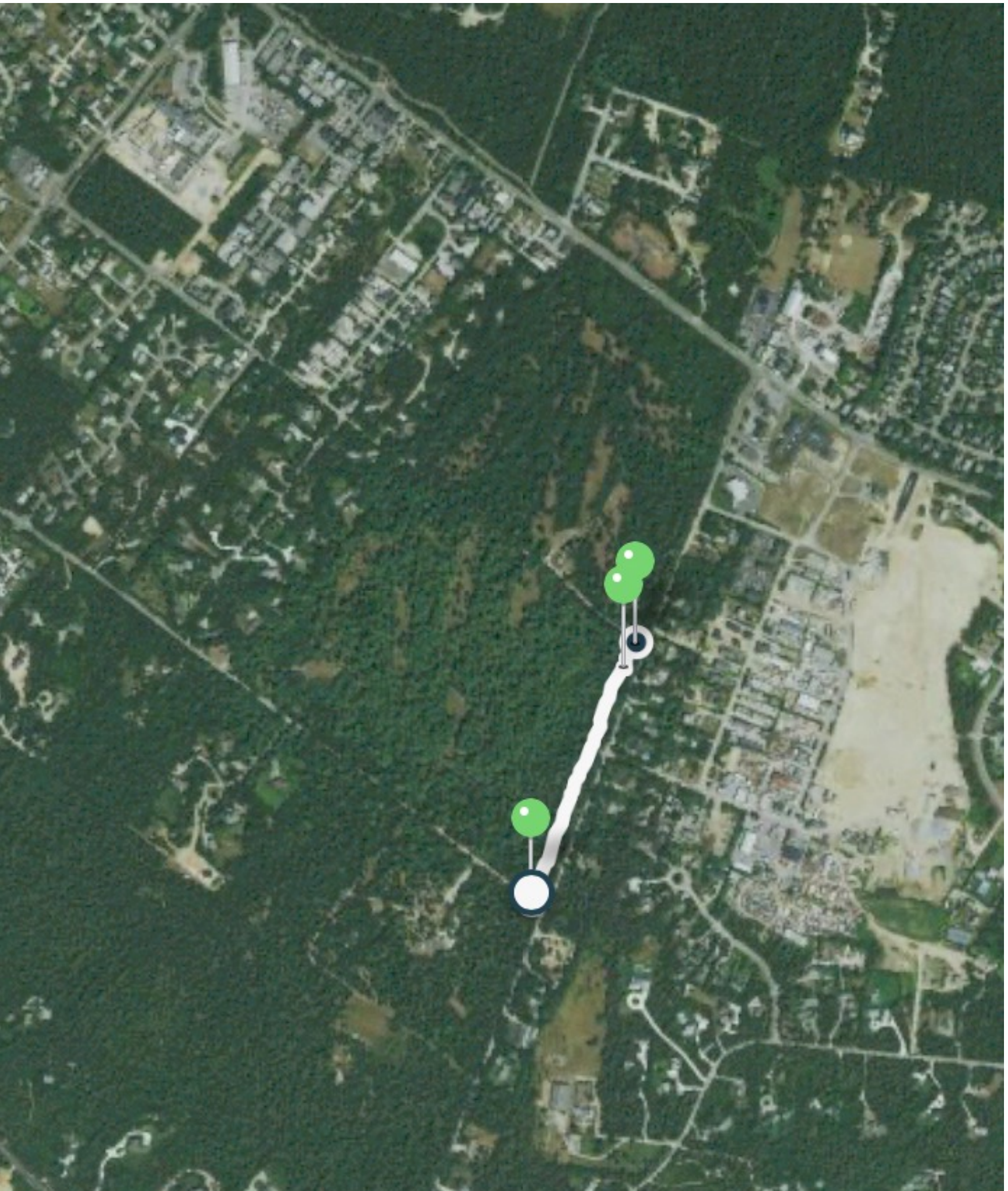

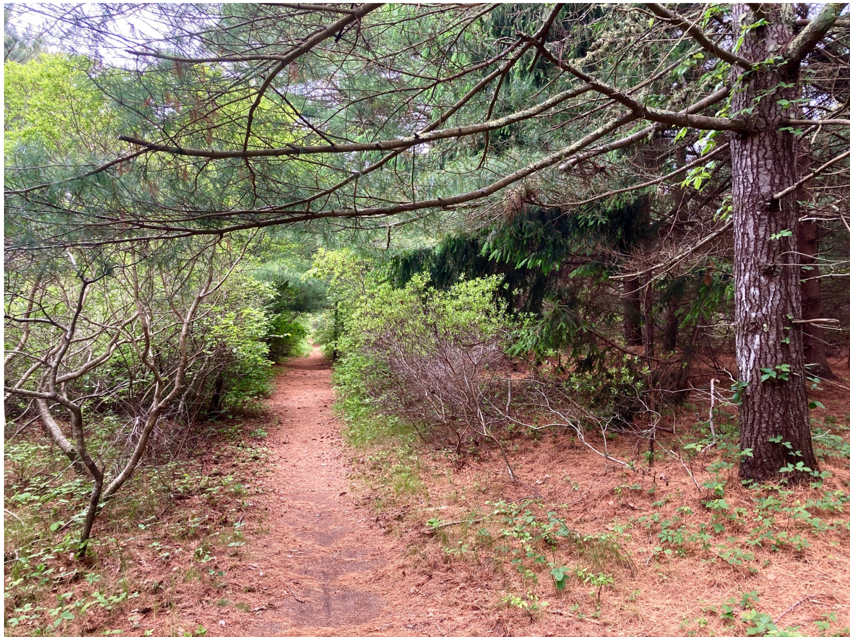

Pine Woods, Water Tower Site – off Polpis Rd.

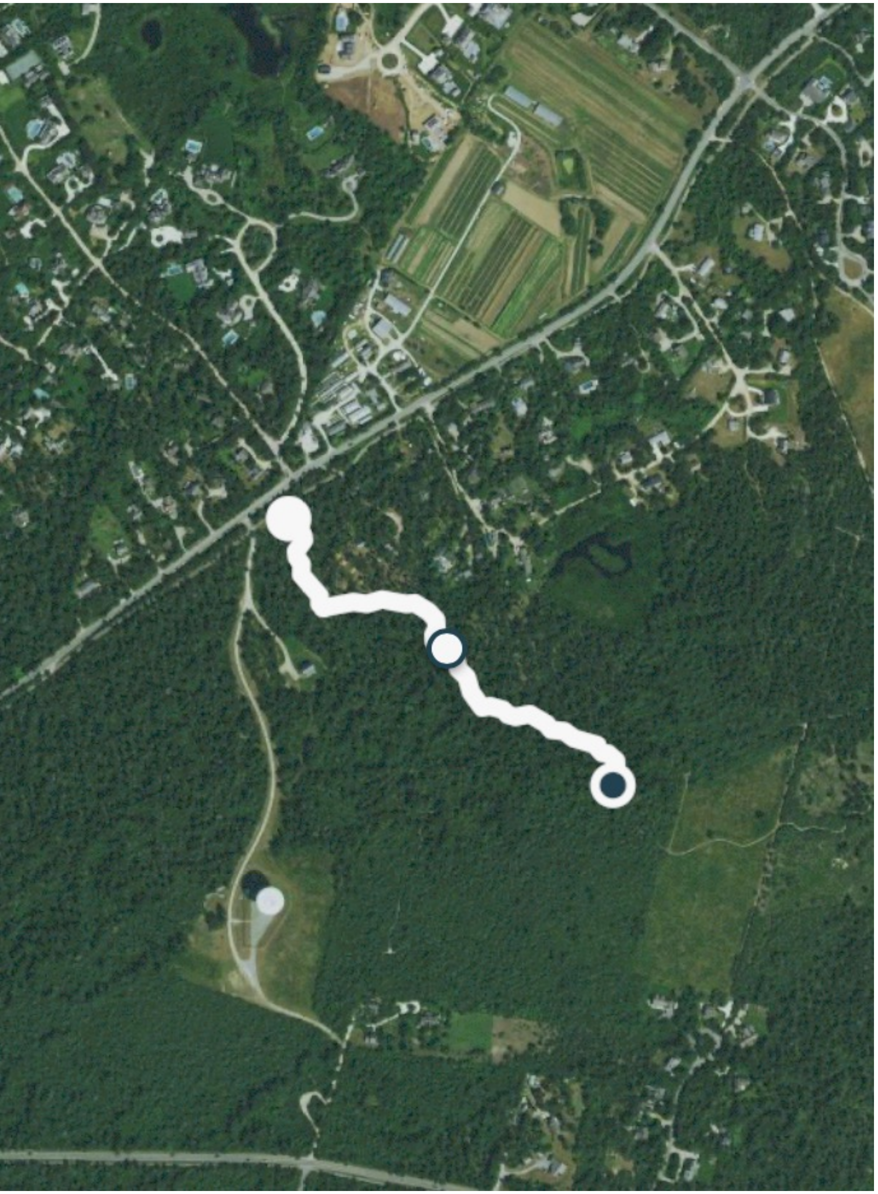

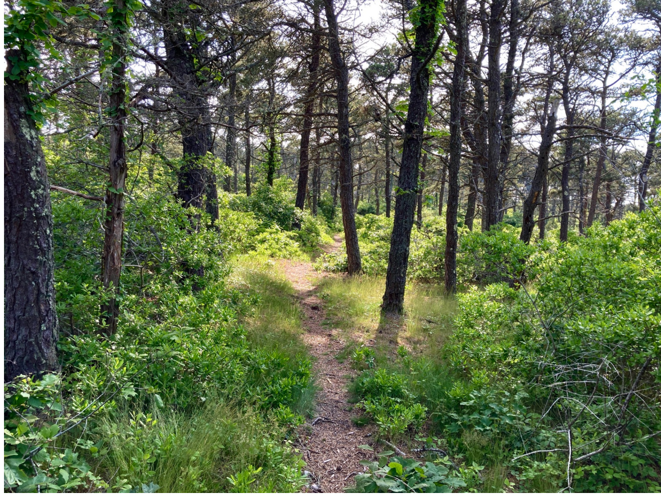

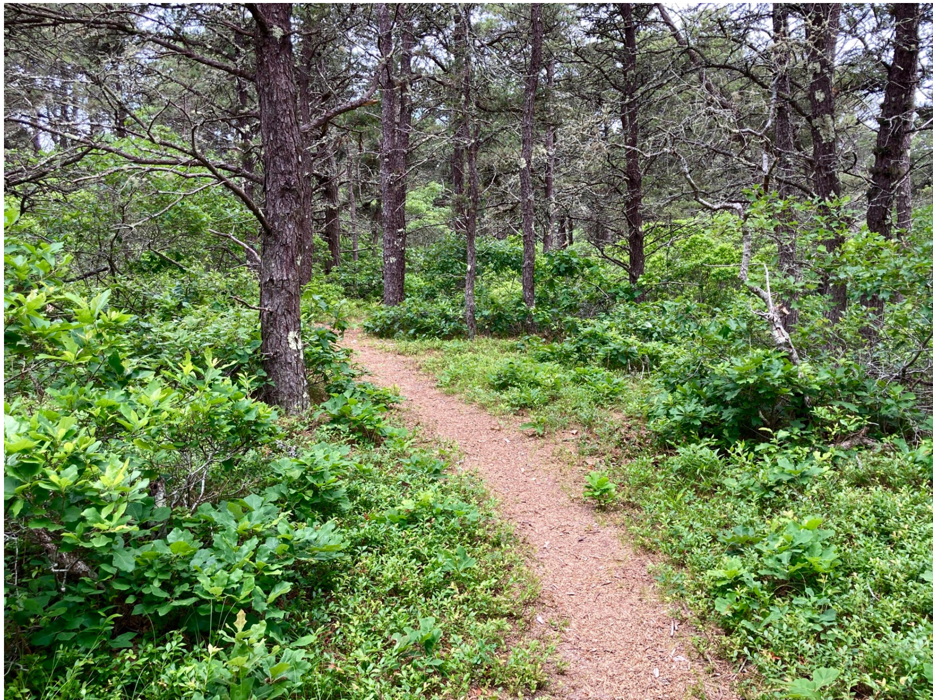

South Pasture Site – off corner of Gloucester St.

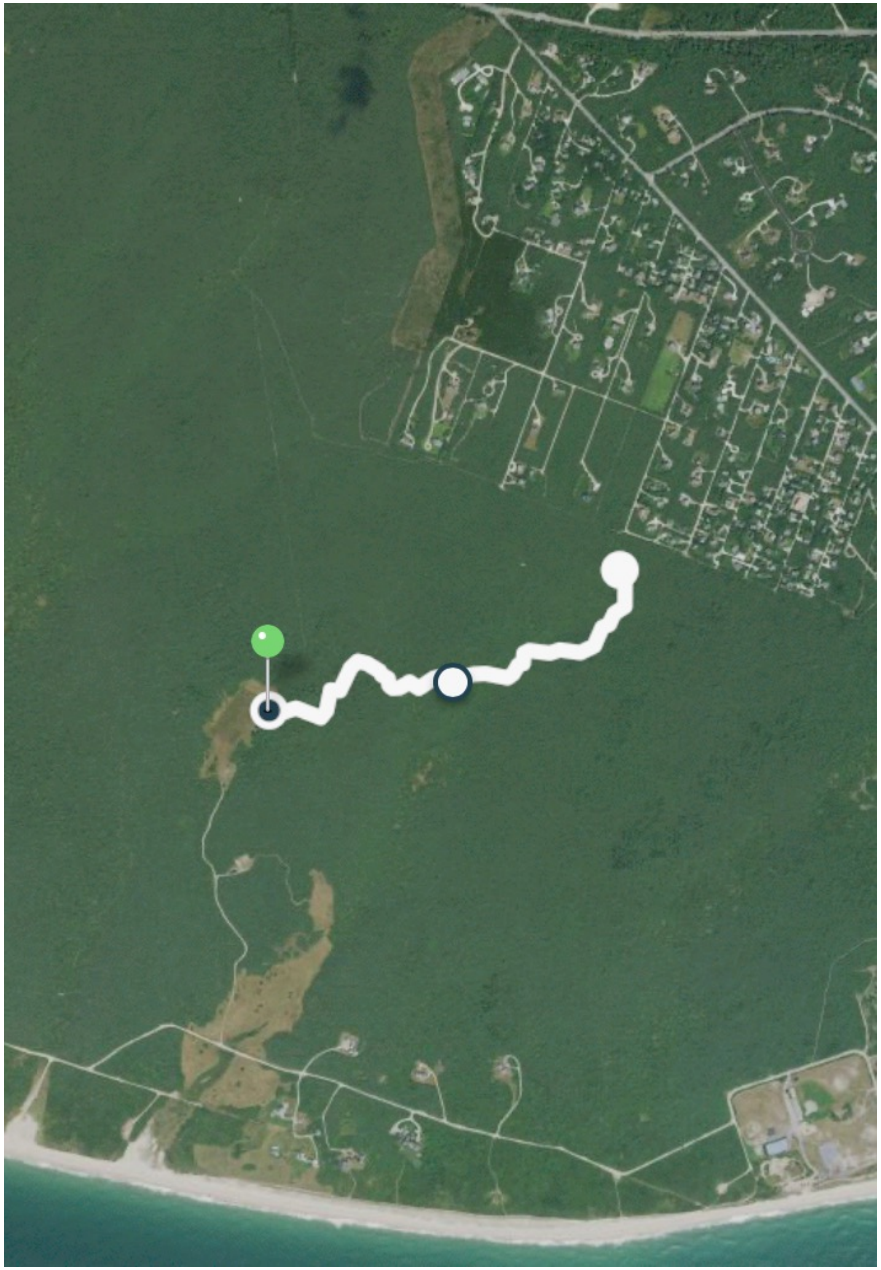

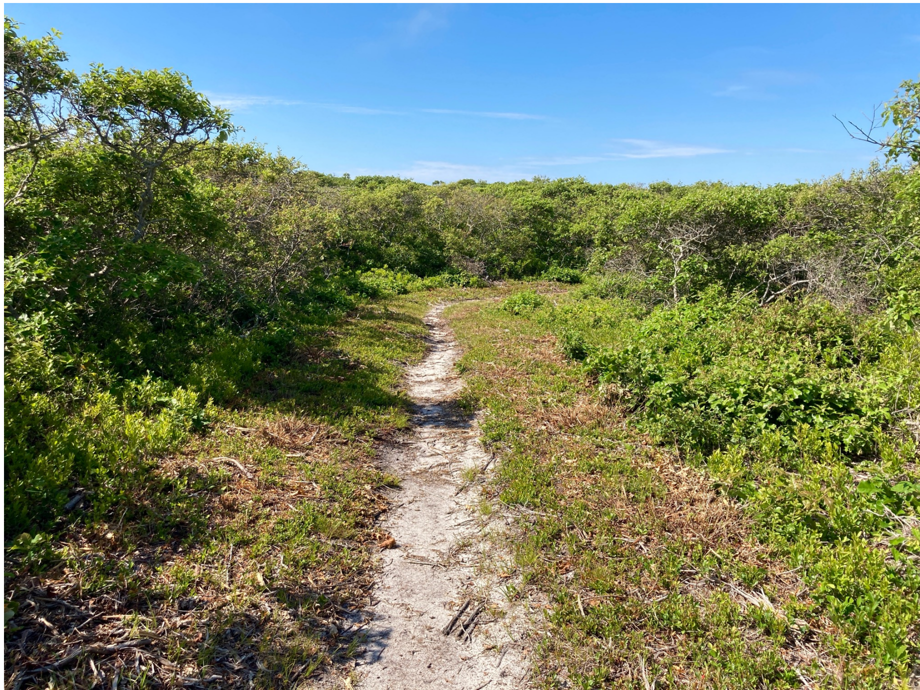

Barrett Farm Road Site – Barrett Farm Rd. near Madaket Rd.

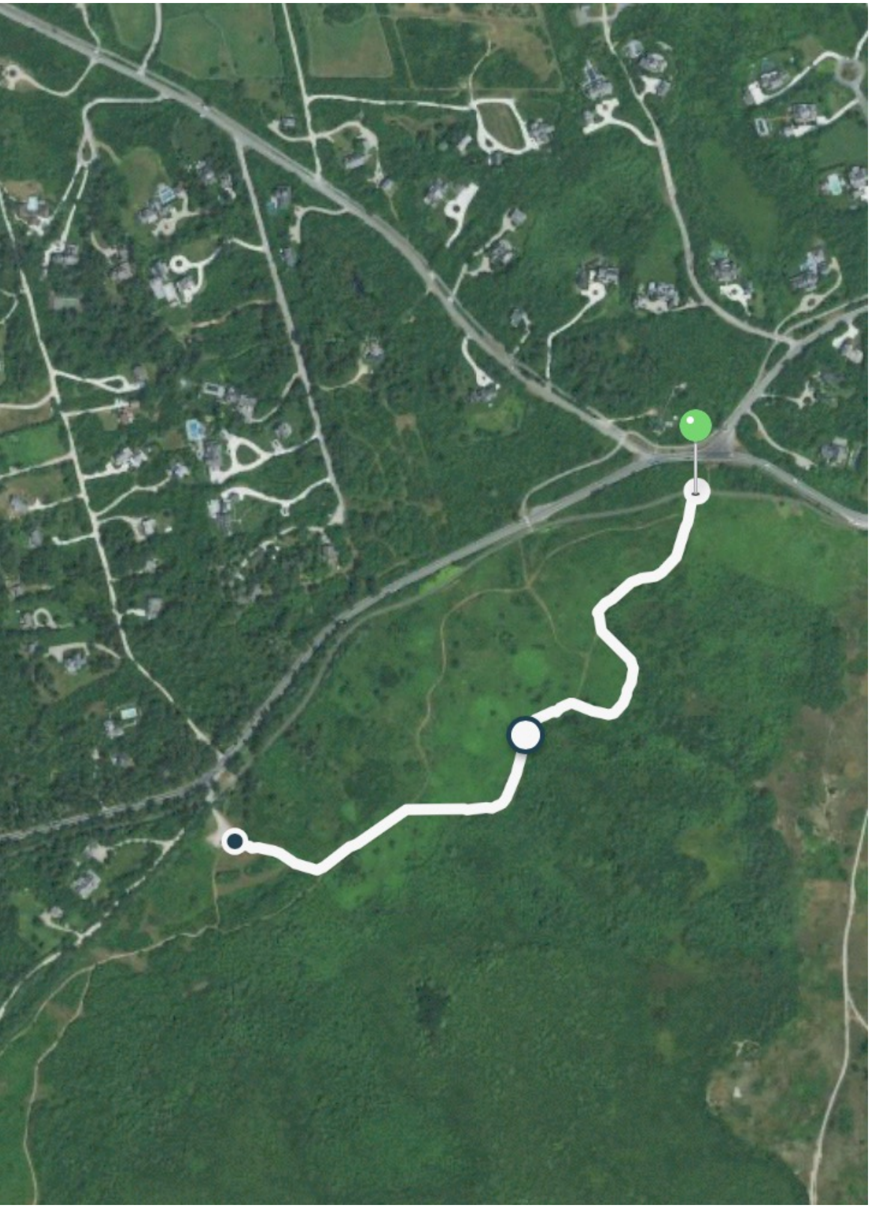

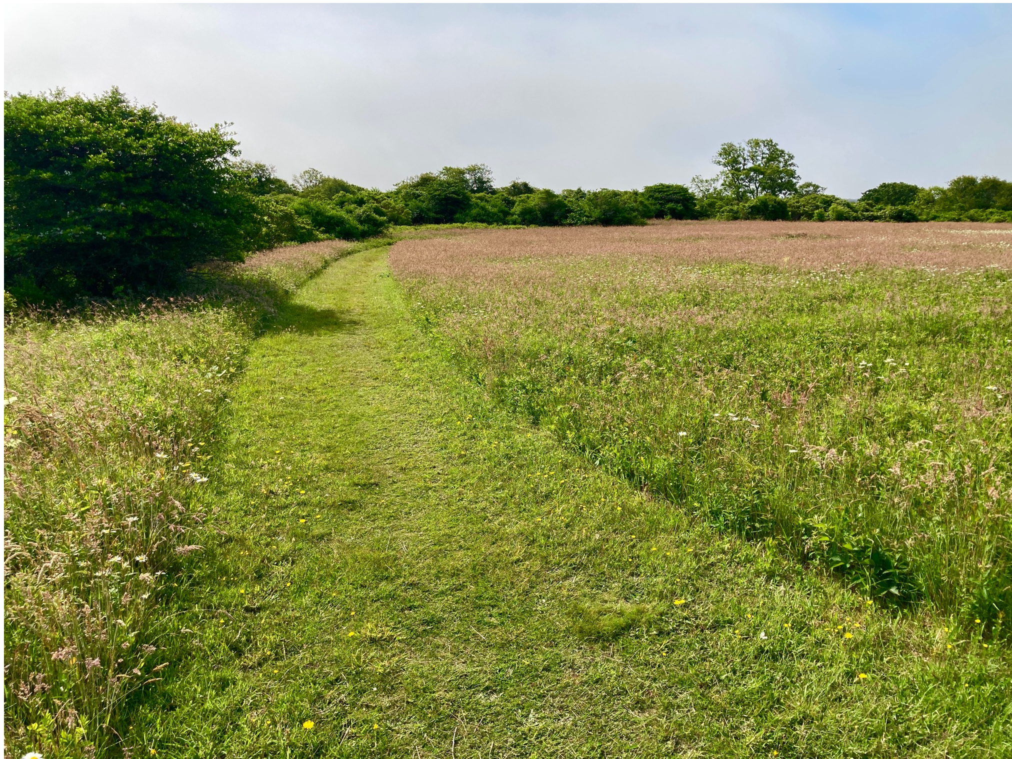

Linda Loring Site – 110 Eel Point Rd.

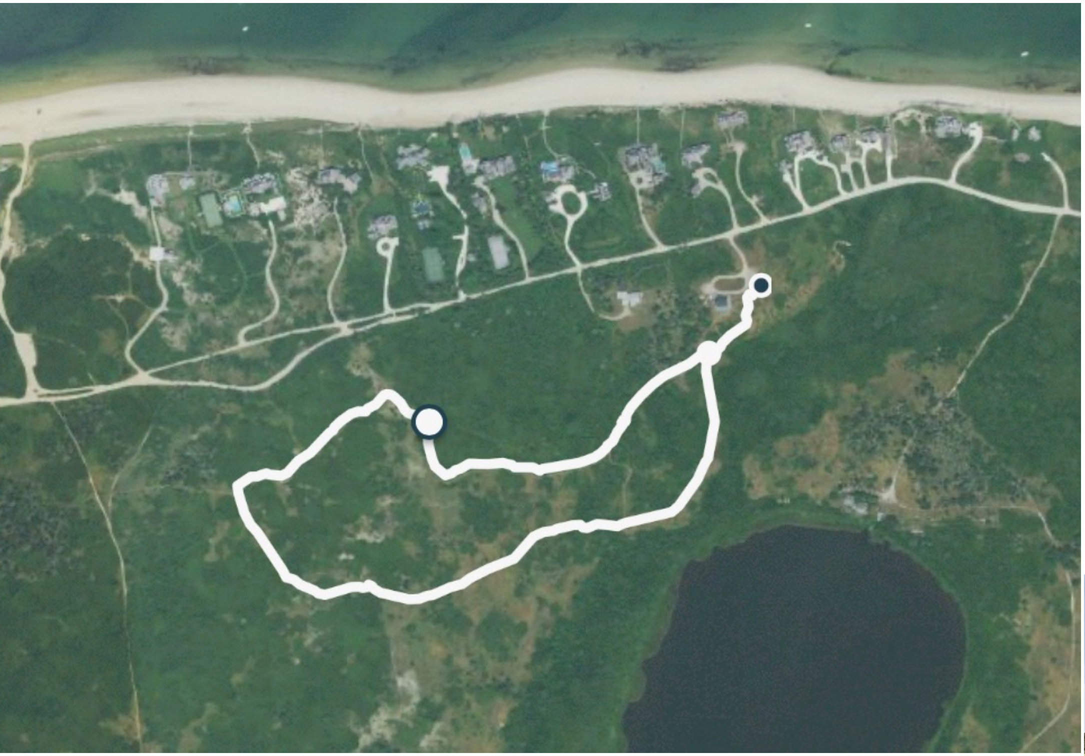

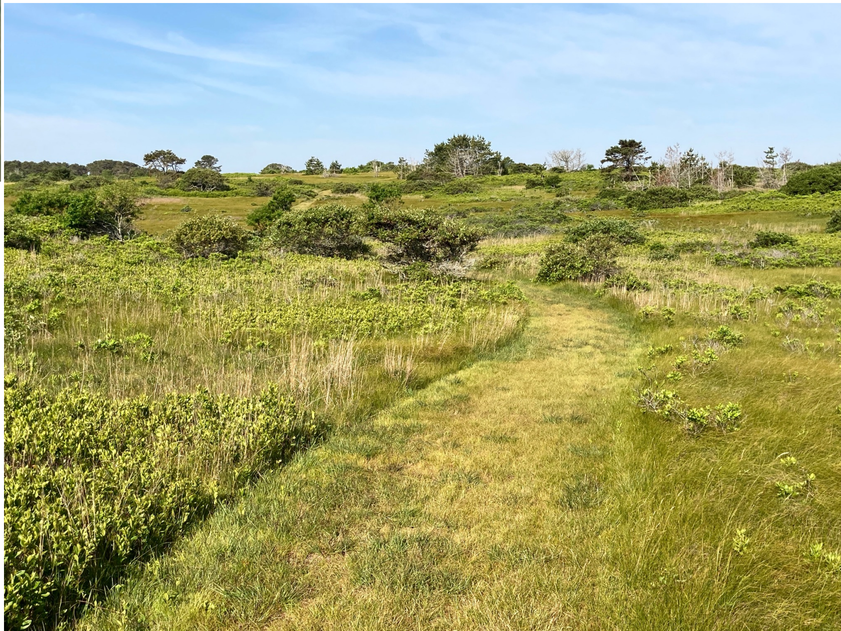

Long Pond (lone star site)

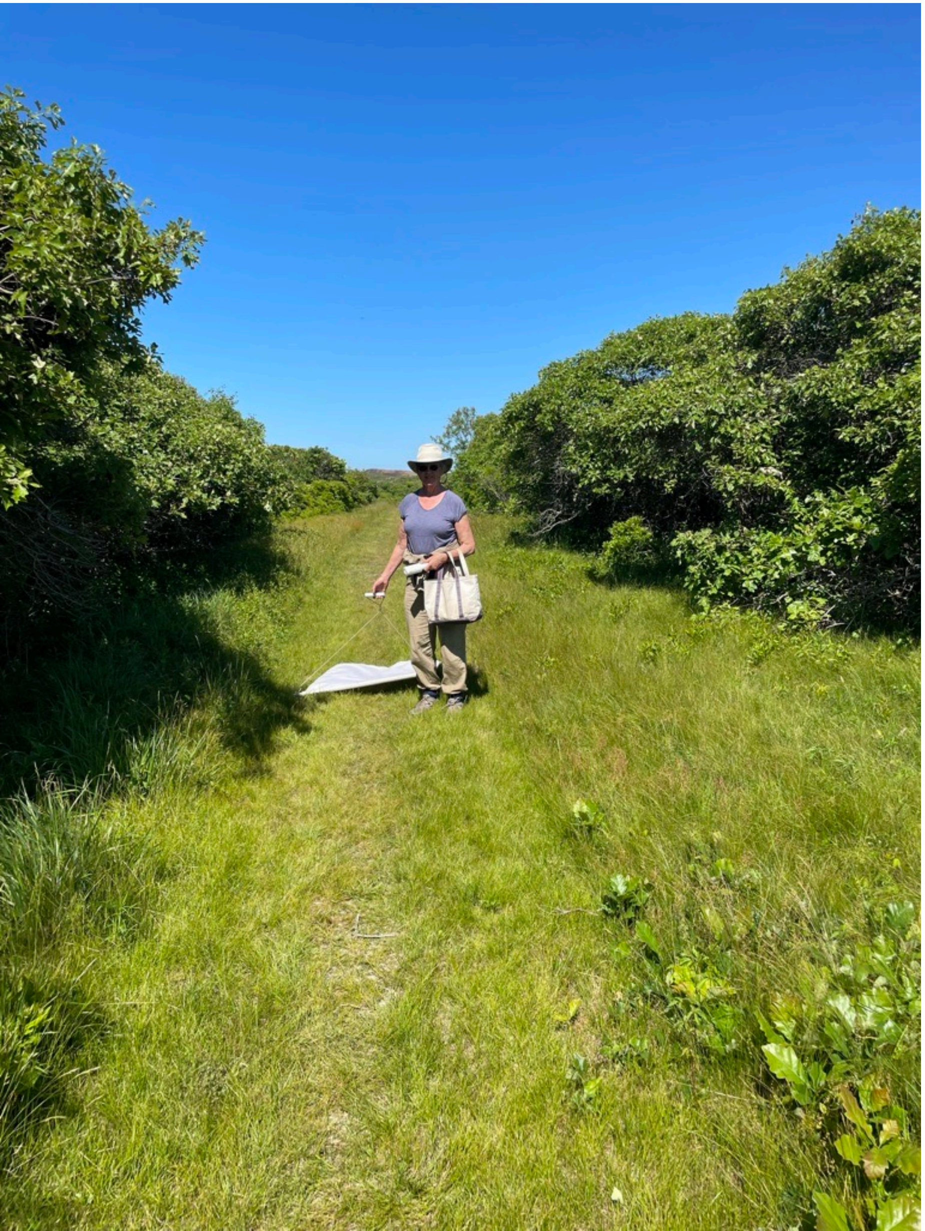

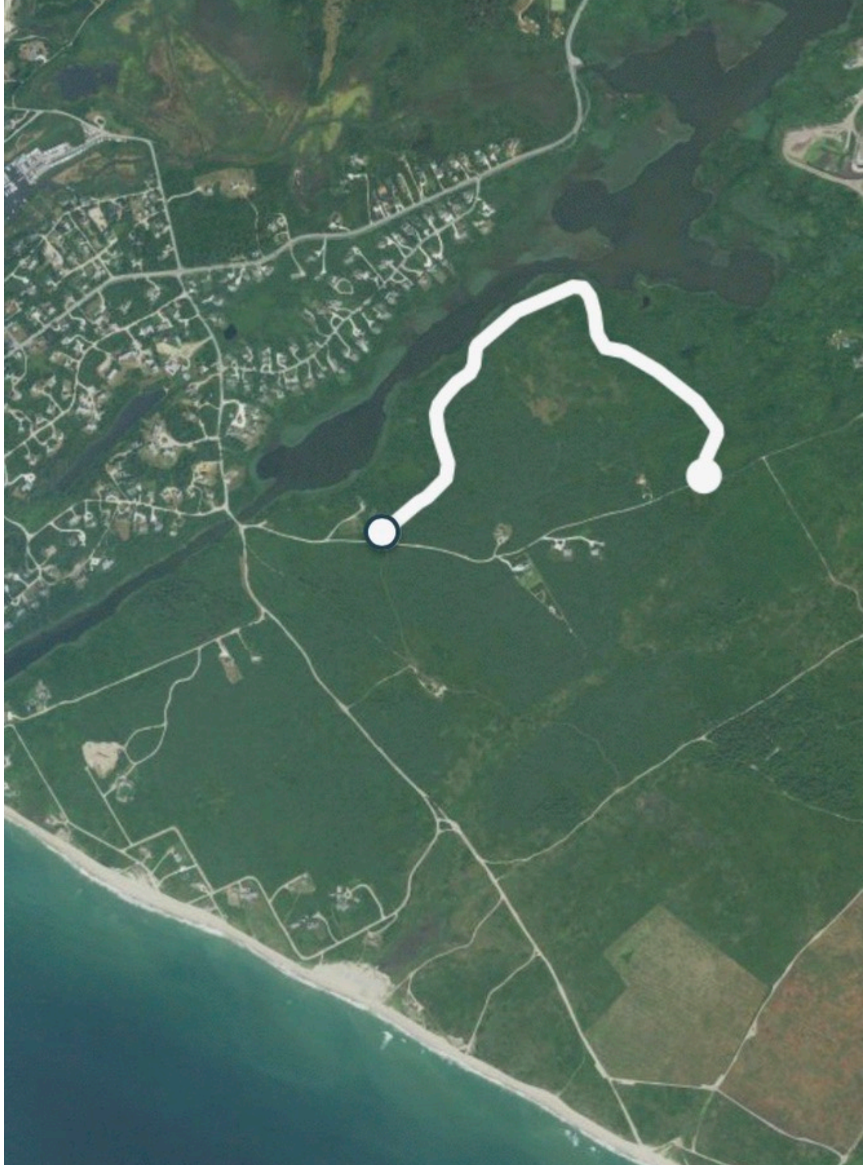

Clark’s Cove (lone star site)

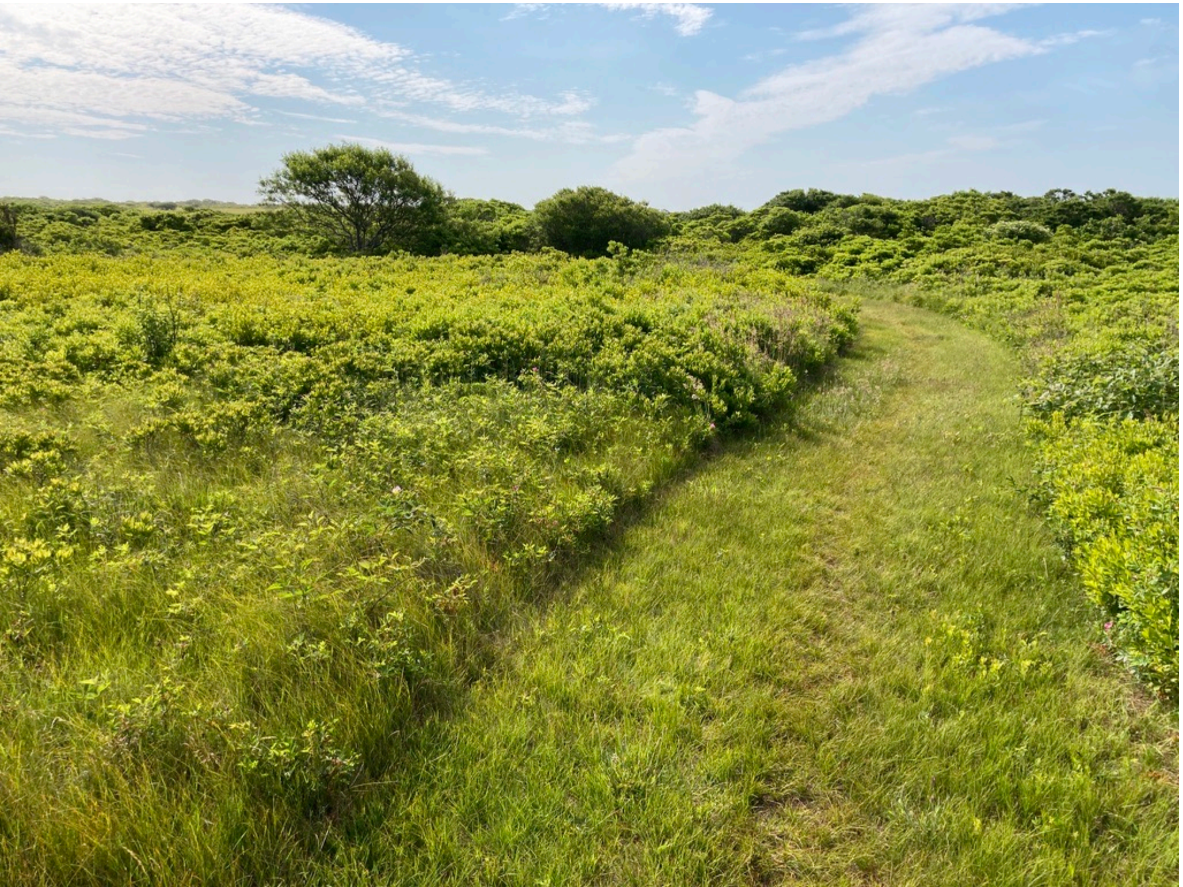

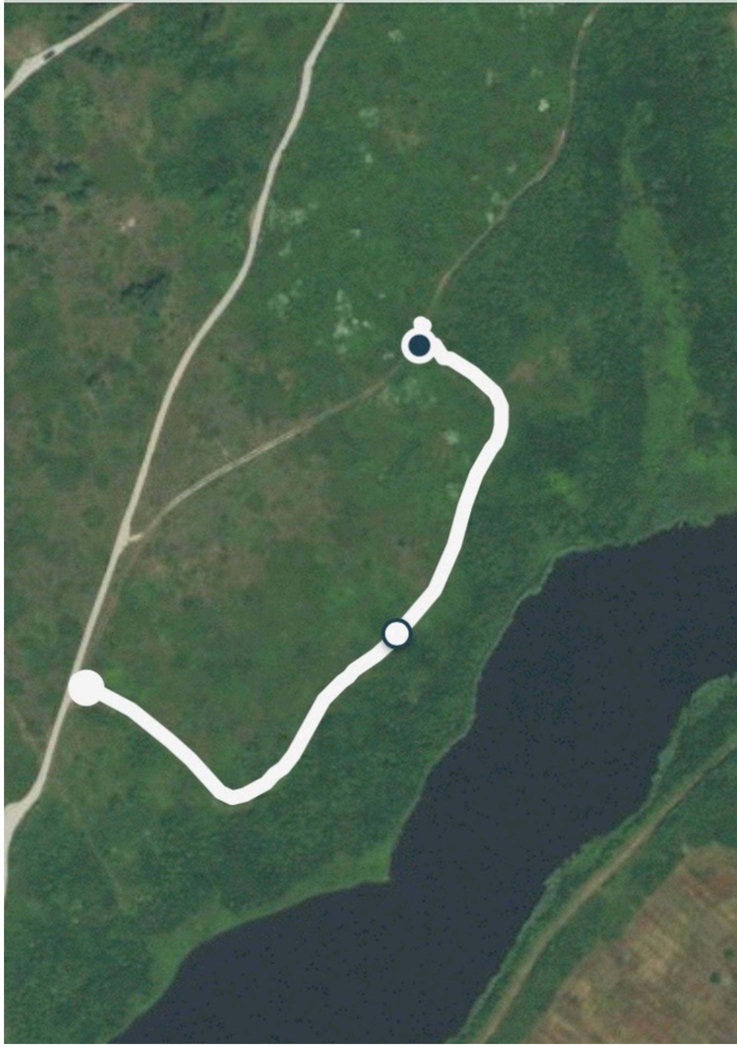

Supplement: Supplementary file 1 [file insects-14-00628-s001.zip › Supplementary Figure S1. Maps and habitat photos of the 12 study sites..pdf]
